# Transcriptional Profiling of Leucocyte Count Variation from Porcine Peripheral Blood Reveals Differential Gene Expression

**DOI:** 10.1155/2018/1496536

**Published:** 2018-11-18

**Authors:** Adeyinka Abiola Adetula, Xiangdong Liu, Thuy Nhien Tran Thi, Ali Akbar Bhuiyan, Xiaoyong Du, Mengjin Zhu, Xinyun Li, Shuhong Zhao, Yunlong Ma, Haiyan Wang

**Affiliations:** ^1^Key Lab of Agricultural Animal Genetics, Breeding, and Reproduction of Ministry of Education, Huazhong Agricultural University, Wuhan 430070, China; ^2^The Cooperative Innovation Center for Sustainable Pig Production, Huazhong Agricultural University, Wuhan 430070, China; ^3^The College of Informatics, Huazhong Agricultural University, Wuhan 430070, China

## Abstract

Leucocytes have tremendous health-check importance related to the individual antiviral capacity of pigs and other mammals. However, the molecular mechanism of the immune response of blood leucocytes in pigs is not completely known. This study investigated the leucocyte-count variation before and after poly I:C stimulation in a Duroc–Erhualian F2 population. Pigs with increased and decreased differences in leucocyte counts were coded as increased responder (IR) and decreased responder (DR), respectively. Then, we used microarray technology to compare the gene-expression profiles of both groups of pigs. Transcriptomic analysis identified 129 differentially expressed genes (DEGs) in IR pigs and 136 DEGs in DR pigs. Forty-one common DEGs showed that both groups had similar expression patterns of immune responses. These results illustrated a differential expression in both groups. Furthermore, qPCR experiment was performed to verify the differential-expression profile. Functional annotation of the DEGs indicated that both IR and DR pigs were similar in several biological processes, including innate immune response, and also exhibited distinct differences in biological processes, molecular function, and pathways. These results provided insights into the mechanism underlying the antiviral capacity of pigs.** Trial registration number** is CAS Registry Number 24939-03-5.

## 1. Introduction

Leucocytes are nucleated blood cells whose rapid translation of mRNA is regulated by signaling events transduced to the cell surface antigen so as to interact with proteins, other cells, and extracellular matrices. Leucocytes are critical in normal pathophysiological processes and acute phase conditions, such as physiologic and metabolic changes that occur in response to generalized acute infections, trauma, severe inflammatory processes, tissue injury, and autoimmune diseases [1]. Leucocytes generated from multipotent, self-renewing progenitor cells develop from mesodermal hemangioblast cells [2]. Immunologists have recognized leucocyte count and leucocyte differential count as key diagnostic measurements because of their sharp increase during acute infections [3]. Therefore, a complete blood cell test often includes a measurement of the level of leucocytes or white blood cells. Peripheral blood contains polymorphonuclear leucocytes that penetrate blood vessel endothelium to enter the area of inflammation where they are essential in destroying the action and increasing the power of resistance against any infection, repair, and regeneration of tissues [2, 4]. Alteration in several immune functions assessed in vitro has been monitored with blood leucocytes obtained from pig and human as key diagnostic measurements during acute infections [3, 5–7]. The characterization of leucocyte surface antigens by monoclonal antibodies and other molecular studies has determined the cell lineages and blood leucocyte subsets implicated in the immune response [8]. Increasingly, investigators have explored the possibility of characterizing the mechanism of diseases by using the subpopulations of leucocytes in vivo, thereby enabling enhanced investigations of the immune response to various porcine infections such as* Actinobacillus pleuropneumoniae*, African swine fever virus (ASFV), classical swine fever virus (CSFV), porcine reproductive and respiratory syndrome virus (PRRSv), and Aujeszky disease virus [9–16]. Strikingly, leucocyte differential count in domestic pig has been associated with multiple chromosomal regions, but specific loci/genes that can partially explain the variation between individuals have not been identified [17–19].

Increased leucocytes are typically found in pigs with conditions associated with viral respiratory tract infection after parainfluenza-3 (PI3) virus [20]. ASFV infection leads to serious changes in the concentration of leucocytes detected 2–3 d after infection [21]. PRRSv infection caused a huge, acute drop in total leucocyte counts that affect all PBMC populations by 2 d after infection in pregnant gilts [22]. To these infectious diseases, the resistance of an individual resulted from both innate and acquired immunity. The capacity of innate immunity or acquired immunity is more or less controlled by genes [23–25]. In pigs, genetic studies have revealed several genes that participate in the resistance to diseases [26] including single-gene porcine ryanodine receptor (ryr1) regulating malignant hyperthermia [27] and porcine factor H [28]. Nevertheless, the identities of the most regulators of leucocytes related traits and their direct targets are largely unknown despite their biological functions.

To define the molecular mechanisms of general gene-expression profiles or changes during rapid leucocytes turnovers in pig genome, we adapted techniques of microarray for genetic profiling of pigs with the increased and decreased leucocyte counts before and after polyinosinic:polycytidylic acid (poly I:C) stimulation. Poly I:C is a synthetic double-stranded RNA that is used experimentally to model viral infections in vivo [29], together with the microarray technology that represents a profiling strategy that allows the detection and measure of various responses by a multitude of gene probes sets. We have identified a number of previously uncharacterized genes that appear to be expressed in swine blood leucocytes, while simultaneously establishing the dominantly expressed genes of the increased and decreased leucocyte phenotypes, as well as immune components involved such as influenza A pathway, cytosolic DNA-sensing pathway, chemokine signaling pathway, and cytokine-cytokine receptor interaction, which are important in the molecular pathways that regulate white cells and/or hematopoietic stem cell function in normal and pathologic conditions.

## 2. Materials and Methods

### 2.1. Ethics Approval

All experimental procedures and animal care activities were strictly conducted in accordance with the guidelines established for the care and use of laboratory animals of the Standing Committee of Hubei People's Congress (No. 5) approved by The Scientific Ethics Committee of Huazhong Agricultural University (HZAUSW-2013-014). Therefore, all efforts were made to minimize suffering.

### 2.2. Animals

Experimental design, details of the infection, and blood collection procedures are described in [30]. The pigs used in the study were from a Duroc–Erhualian F2 population and comprised 392 F2 offspring derived from 51 F1 and 26 F0 parents. In short, 8 Duroc boars were mated to 18 Erhualian sows. Then, 13 F1 boars and 38 F1 sows were chosen and mated to produce 392 F2 animals. The six animals used in the present study were selected based on the two-tailed value of leucocyte counts before and after poly I:C stimulation. All animals are fed in the same conditions and free of virus infections such as PRRSv,* Mycoplasma hyopneumoniae*, and swine influenza virus. At 35 d, pigs were intramuscularly infected with poly I:C (CAS Registry Number 24939-03-5, Hangzhou Meiya Pharmacy) at a dose of 0.5 mg/kg. Blood samples were taken at 33 and 35 d after poly (I:C) infection. Leucocytes levels on these dpi were measured using a Japanese photoelectric MEK-8222K automatic 5 classification blood analyzer (Nihon kohden, Tokyo, Japan).

### 2.3. Phenotypic Data

The statistical significance of the phenotypic differences between the treatment and control groups was first examined by assuming unequal variances Student's t-test. The residuals obtained from the linear model were further used as a target variable for analysis using t-test and lm functions in the R environment.

### 2.4. Microarray Design and Hybridization

Based on leucocyte count recorded at 33 and 35 d after poly (I:C) infection, a total of six pigs were divided into two groups and coded as increased responders (IR) and decreased responders (DR). Whole blood samples were collected from each of the six pigs 4 h after poly I:C treatment on day 35 and at day 33 as an unstimulated control. The whole blood samples were used for the microarray experiments. GeneChip Porcine Genome Arrays (Affymetrix) were used to determine gene expression levels in the whole blood samples before and after poly I:C treatment of the six pigs. RNA labeling and hybridization were performed by a commercial Affymetrix array service (GeneTech Biotechnology Limited Company, Shanghai, China). This methodology was described in our previous study [31]. All raw probe-microarray data were normalized by the Robust Multichip Average method from packages of Bioconductor (http://www.bioconductor.org) implemented in the R environment [32–34]. The model matrix for differentially expressed genes (DEGs) identification was defined in a linear format, and the model scenario included the same factors in the linear model for leucocyte count phenotype analysis. The linear models for microarray data (LIMMA) [35] were used to identify the DEGs. To determine the statistical significance of the DEGs, the cutoff for P values was set to (0.01), adjusted P values (0.1), and fold change ≥ (2.0). Furthermore, a two-way hierarchical clustering analysis was performed to identify the DEGs patterns according to [35].

### 2.5. Gene Annotation and Pathway Analysis

The Affymetrix porcine genome microarray annotation was initially performed using the annotation file given by Affymetrix, Inc. (https://www.affymetrix.com/index.affx). Each probe set was annotated using the method previously described by [36]. The differentially expressed transcripts were further compared with the official homologous human gene symbols. Using bioinformatics resource tools in the DAVID website v6.8 [37, 38], differentially expressed genes (DEGs) were uploaded to determine the regulated pathways, and biological function significantly associated with the gene lists. Therefore, this statistical test assesses the proportion of genes that map to a particular function or pathway in the IR and DR phenotypes. We focused on the most affected pathways, biological, molecular, and cellular functions that belonged to the two phenotypes gene symbols were used in the subsequent functional analysis.

### 2.6. Validation of DEGs by qPCR

Quantitative real time-PCR (qPCR) was performed to quantify the expression levels of 14 DEGs (ISG20, RSAD2, TLR4, S100A12, S100A9, S100A8, SH2DIA, TNK2, MX1, MX2, OAS1, PLSCR1, tripartite motif (TRIM) 26, and STAT1). One microgram of total RNA obtained from all biological replicates (3 by 3 replicates of IR and DR, before and after poly I:C stimulation) was reverse-transcribed using EasyScript™ one-step gDNA Removal and cDNA Synthesis SuperMix (TansGen Biotech, Beijing, China) following manufacturer's protocols for the two-step qPCR assays. Quantitative PCR was then performed by using SYBR qPCR Mix (Aid lab Biotechnologies Co., Ltd., China) in Bio-Rad thermal cycler, CFX-384, real-time system. Details of genes, annotations, and primer set validated by qPCR are included in Supplementary File 1. Following amplification, the differences in the Ct values of the control and experimental samples were used to determine the relative expression of the gene in each sample. All Ct values were normalized using *β*-actin gene [39, 40]. The relative gene expression levels were calculated using 2−(ΔΔCT) method [41, 42]. The correlation between the microarray and qPCR results for the gene set was then performed for each replicate and the statistical significance of the correlations determined. The log2 fold-change of microarray versus qPCR log2 fold-change was graphed to determine the quality of correlation; a slope of one would define perfect correlation. The strength of the relationship was quantified by Pearson's coefficient computation.

## 3. Results

### 3.1. Poly I:C-Stimulated Leucocyte-Count Variation

**Figure 1:**
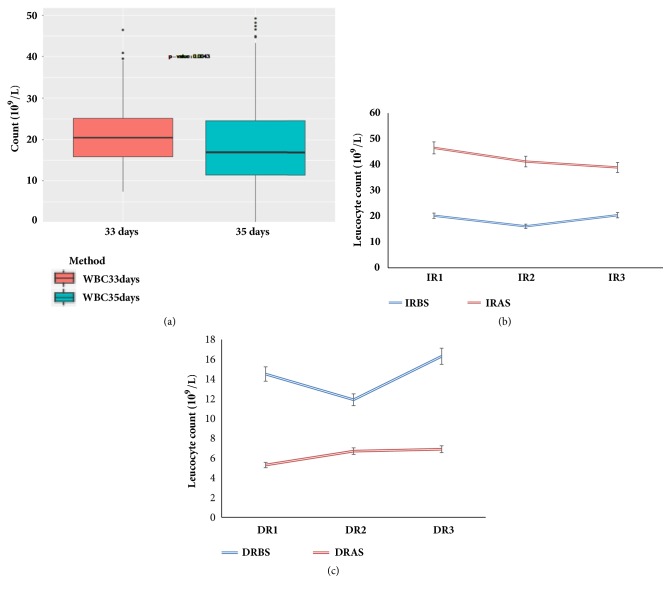
Changes in (a) poly I:C-induced peripheral blood leucocyte. (b) The leucocyte count changes in IR (IR1, IR2, and IR3); IRBS/IRAS represent IR before and after poly I:C stimulation. (c) Leucocyte count changes in DR (DR1, DR2, and DR3); DRBS/DRAS represent DR before and after poly I:C stimulation. Error bars indicate standard error of mean (SEM).

Potent immune stimulator led to significant pronounced differences in leucocyte counts. Overall mean leucocyte counts were found to have a huge variation before and after poly I:C stimulation (p < 0.05) (Figure 1(a)). Before stimulation, the overall mean leucocyte-count values (Mean ± SEM) were 21.15 ± 1.27; after stimulation, overall mean leucocyte-count values were 19.10 ± 1.15 (n = 277) (Figure 1(a)). However, the mean leucocyte-count values of IR and of DR were calculated from the two-tailed critical value of leucocyte counts after 4 h poly I:C treatment. The mean leucocyte-count values for IR group were 18.90 ± 10.91 before treatment and after treatment were increased to 42.20 ± 24.36 (Figure 1(b)). By contrast, the DR leucocyte-count values were 14.23 ± 8.22 before treatment and after treatment were reduced to 6.30 ± 3.64 (Figure 1(c)). Increased leucocyte count may also explain their ability to fight back and persist in response to viral infection (Figure 1(b)), whereas a significant decrease in leucocyte count suggested that poly I:C temporarily disrupts bone marrow function where leucocytes are made (Figure 1(c)). The differences between the two groups were statistically significant (p < 0.01) in the IR and DR groups, respectively.

### 3.2. Transcriptome Clustering and Altered DEGs between IR and DR

**Figure 2:**
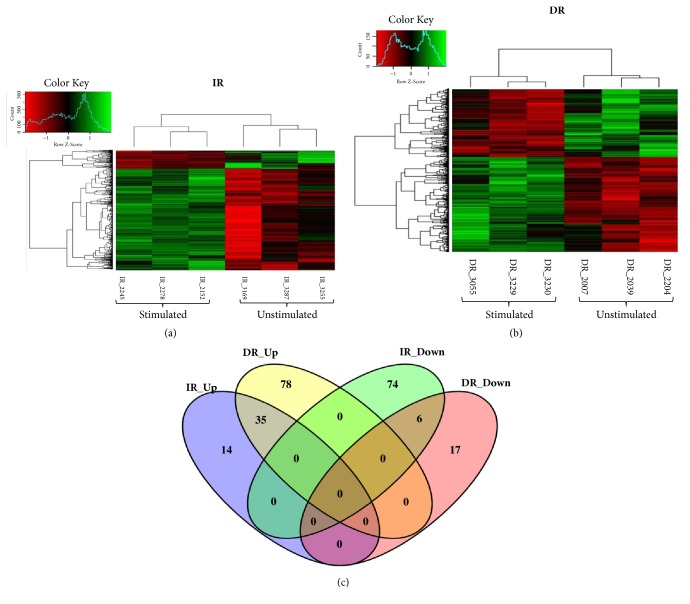
Two-way hierarchical clustering and Venn diagram of overlapped DEGs by poly I:C stimulation. Gene expression values in (a) IR pigs and (b) DR pigs. The columns within the heat-map represent samples (pigs ID) while the rows represent genes. The color scheme red (high) expression and green (low) expression. The color legend at the top-left of the figure indicates the fold change in gene expression. (c) The Venn diagram of DEGs in the two groups.

To understand further the patterns of differential gene expression and also to know whether the poly I:C stimulation effected transcriptionally in the selected pigs, two-way hierarchical clustering was conducted with DEGs for the construction of heat-map. The heat-map illustrates gene expression values between the IR (Figure 2(a)) and DR (Figure 2(b)). The patterns of gene expression indicate that all the samples clustered into two types indicating pre- and post-poly I:C stimulation. Thus, to compare temporarily the transcriptome altering between the IR and DR pigs post-poly I:C treatment, we further analyzed the DEGs in both groups and asked how many of the DEGs overlapped and/or were differentially expressed specifically in each phenotype (Figure 2(c)). 129 DEGs were found in the IR group and 136 DEGs in the DR group (Supplementary File 2). Notably, many DEGs have been reported to be involved in the immune regulatory processes (Table 1). A total of 35 upregulated and 6 downregulated overlapping DEGs were found in both groups. The IR has 14 and 74 unique upregulated and downregulated DEGs, respectively, and the DR has 78 and 17 unique upregulated and downregulated DEGs, respectively (Figure 2(c), Supplementary File 2). Following those findings, more DEGs were downregulated in the IR pigs than in the DR pigs, thereby indicating that IR pigs showed less responsiveness at the transcription level than did the DR pigs.

### 3.3. Gene Expression Verification of DEGs with qPCR

**Figure 3:**
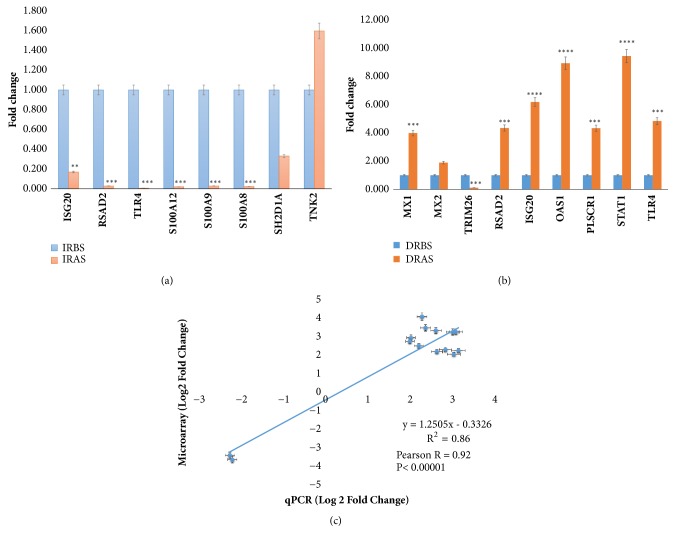
Microarray fold change verification with qPCR. (a, b) Fold change of 14 genes of IR and DR, before and after stimulation coded as IRBS/IRAS and DRBS/DRAS, respectively. Data are reported for three technical replicates in all biological samples. Error bars indicate standard error of the mean (SEM). Each histogram represents the level of the target gene relative expression level. Asterisks symbolized P value significance (p ≤ 0.05 (*∗*), p ≤ 0.01 (*∗∗*), p ≤ 0.001 (*∗∗∗*), and p ≤ 0.0001 (*∗∗∗∗*) based on assuming unequal variances Student's t-test. (c) qPCR validated 14 genes. A line plot of qPCR log2 (FC) and microarray log2 (FC) data of 14 genes. The linear relationship between the qPCR and microarray log2 (FC) data based on the R^2^ value, and the line linear regression (Y).

A total of 14 genes of biological interest identified by the microarray were verified by qPCR. The tested genes encoded enzymes involved in interferon-stimulation, transcription factors, transmembrane and receptor binding, regulation of ubiquitin-protein ligase, cellular activity, and lymphocyte-activating from different gene families and molecules playing a role in the immune response of IR and DR. Relative expression of 6 upregulated genes (ISG20, RSAD2, TLR4, S100A12, S100A9, and S100A8) was significantly reduced after poly I:C stimulation in the IR. Downregulated gene (SH2DIA) expression was reduced after poly I:C stimulation and also downregulated (TNK2) gene expression increased after poly I:C stimulation in the IR (Figure 3(a)). Eight upregulated genes, namely, MX1, MX2, RSAD2, ISG20, OAS1, PLSCR1, STAT1, and TLR4, were significantly reduced before poly I:C stimulation and increased after poly I:C stimulation in the DR. Upregulated gene (TRIM26) expression was significantly increased before poly I:C stimulation and reduced after poly I:C stimulation in DR (Figure 3(b)). Additionally, we found strong agreement between qPCR and microarray for the fourteen genes verified. The results were presented in fitted line plot and correlation output suggests a positive linear relationship between qPCR and microarray log2 fold-change. The value of R^2^, the coefficient of determination, was 0.86; and the linear regression model Y = (1.2505x – 0.3326) and the Pearson correlation was 0.92, close to one. All genes expression profiling experiments were consistent with the expression profiles determined from microarray data (Figure 3(c)).

### 3.4. Comparative Functional Annotation of Poly I:C Induced DEGs between IR and DR

**Figure 4:**
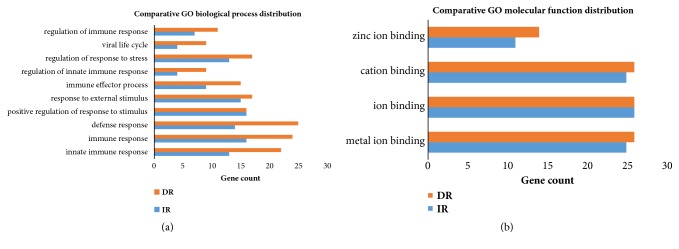
Commonly enriched gene ontology (functional classification) of the DEGs by poly I:C stimulation in IR and DR. (a) Comparative GO biological process distribution of the DEGs by poly I:C stimulation in the IR and DR pigs. (b) Comparative GO molecular function distribution of DEGs by poly I:C stimulation in the IR and DR pigs.

Consider a total of 265 DEGs in the IR and DR phenotypes (Log2 FC ≥ 2 and ≤-2.0) and 201 orthologues of human genes were annotated in the DAVID (Database for Annotation, Visualization, and Integrated Discovery) database, which revealed 20 significant functional annotation clusters between the IR and DR (p < 0.05). The most significant clusters were presented in Table 2. The functional annotation clusters in the IR and DR were related to innate immunity, innate immune response, antiviral defense, immunity, and defense response to the virus but differ in the negative regulation of viral genome replication (Table 2). Furthermore, the DEGs of IR and DR in biological processes (BP), molecular functions (MF), and cellular components (CC) were revealed based on the GO categories in DAVID. When considering the commonly shared biological processes between IR and DR phenotypes, these include innate immune response, immune response, defense response, response to external stimulus, immune effector process, and viral life cycle (p < 0.05 and count ≥ 4) (Figure 4(a), Supplementary File 3). For the GO molecular function, only four terms were common between the IR and DR, including metal ion binding, ion binding, cation binding, and zinc ion binding, whereas the majority of the MF terms were related to the DR background (Figure 4(b), Supplementary File 3). In the cellular component category, DR was distributed mostly in extracellular exosome, extracellular vesicle, extracellular organelle, and extracellular region, whereas no annotation information was found for the IR (Supplementary File 3).

Based on conserved orthologues defined by the Kyoto Encyclopedia of Genes and Genomes (KEGG), the DEGs of IR and DR were assigned to one or more conserved biological pathways. The pathways in KEGG and related genes found in DR were listed in Table 3, whereas no pathway annotation record was found for the IR. The analysis identified nine pathways in DR including influenza A, measles, herpes simplex infection, cytosolic DNA-sensing pathway, hepatitis C, hepatitis B, chemokine signaling pathway, and cytokine-cytokine receptor interaction pathway (Table 3, Supplementary File 4). The pathway of influenza A was the most enriched pathway related to the upregulated genes of DR. Up- and downregulated genes of DR including RNASEL, TNFSF10, OAS1, ZBP1, MX1, CCL8, GNB4, PAK1, STAT1, and IL13RA1 were also enriched in the chemokine signaling and cytokine-cytokine receptor interaction pathways except for upregulated ISG15 in the RIG-I-like receptor signaling pathway. The DR had more DEGs related to the affected pathways when compared with IR.  Table 4 shows the DEGs between IR and DR in the affected pathways.

## 4. Discussion

### 4.1. DEGs Specific to the IR and DR Pigs

In the DEGs specific to the IR pigs, 88 genes were detected; many of these genes were significantly enriched, and some of them had a putative functional role relevant to innate immune response and leucocyte migration involved in inflammatory response. We selected four genes that play roles in the cellular process and signaling. Among cellular process genes, a set of calcium-binding proteins were identified in the IR, including S100A12, S100A9, and S100A8. These genes encode S100 proteins. In the IR group, S100A12, S100A9, and S100A8 were highly expressed before poly I:C stimulation and significantly lower expression was observed after stimulation. These calcium-binding protein genes, considered markers at the site of inflammation as previously reported in humans [51] and pigs [31, 52], play a major role during infection. Among signaling genes, SH2D1A encodes SH2 domain-containing protein 1A of 128 amino acid (aa) residues (Genbank NC_010461.5.). Reference [53] found that SH2D1A interacts with signaling lymphocytic activation molecule (SLAM), thereby demonstrating its contribution to the regulation of a transmembrane protein that is expressed on the surface of activated T and B cells. The expression of SH2D1A reduced after stimulation compared with control in IR; we speculate that it may participate in activating the host immune response during infection.

Considering the 95 DEGs specific to the DR pigs, all the genes that fell within this category had defined functions. Nevertheless, we noticed six genes, including TRIM26, OAS1, STAT1, PLSCR1, MX1, and MX2, whose functional roles can regulate host immune responses. TRIM26, which encoded a member of the TRIM family implicated in innate and adaptive immunity [54, 55], was significantly lower after poly I:C stimulation compared with control in the DR pigs. OAS1 belongs to the OAS family of proteins known to be active synthetases that synthesize 2′-5′-linked oligoadenylates in response to viral infections, thereby affecting an early step of the viral replication cycle [56, 57]. The expression of OAS1 was significantly higher after poly I:C stimulation in DR pigs when compared with control. For STAT1, a critical regulator of the interferon signaling pathway [58, 59], its expression was significantly higher after poly I:C stimulation in the DR compared with control. Phospholipid scramblase 1 gene (PLSCR1) was highly significant after poly I:C stimulation compared with control. Studies in human reported PLSCR1 gene in the regulation of phosphatidylserine distribution of erythrocytes [60]. Moreover, MX1 and MX2 genes were highly expressed in DR pigs after poly I:C stimulation. MX dynamin-like GTPase 1 (MX1) was implicated in some viral replications, including CSFV [61] and pig-original bovine viral diarrhea virus 2 (BVDV-2) [62]. Additionally, myxovirus (influenza virus) resistance 2 gene (MX2), which encodes interferon (IFN)-inducible proteins, plays a critical role in the antivirus state [63, 64]. Similarly, [6] reported the expression of MX1 and STAT1 genes were highly expressed shortly after experimental influenza A virus (IAV) infection in circulating leucocytes.

### 4.2. Biological Processes Found in the IR and DR Phenotypes

For the common biological processes involved in the IR and DR phenotypes, innate immune response, immune response, defense response, response to external stimulus, and immune effector process were the most enriched biological functions found in the IR and DR phenotypes. Among these biological processes, upregulated genes (radical S-adenosyl methionine domain containing 2, interferon stimulated exonuclease gene 20, and DExD/H-box helicase 58) were significantly enriched. Chemokine (C-C motif) ligand 8, SH2 domain contained 1A, tyrosine kinase non-receptor 2, and interleukin 7 receptor were overrepresented downregulated genes regulating the biological function of the IR and DR phenotypes. Additionally, DR phenotype had more transcriptional genes than did the IR in most of the common biological pathways, thereby suggesting that the former created more effective host immune responses than did the latter. Moreover, IR and DR phenotypes differ in several biological defenses, such as cell motility and cellular protein modification process being found in IR phenotype, whereas the regulation of molecular function, positive regulation of cellular metabolic process, and response to cytokine are event-activated for the host immune responses in DR phenotype.

### 4.3. Pathway Regulated by the DEGs in the DR Pigs

In the IR pigs, no pathways were detected. However, several pathways related to antiviral responses were found in the DR pigs, such as influenza A, chemokine signaling pathway, cytokine–cytokine receptor interaction, and RIG-I-like receptor signaling pathway.

Research reports indicate that the influenza A pathway was known to activate cellular signal transduction pathways such as NF-*κ*B signaling, PI3K/Akt pathway, MAPK pathway, PKC/PKR signaling, and TLR/RIG-I signaling cascades [65]. These pathways are important for viral entry, viral replication, viral propagation, and apoptosis; these pathways are involved in antagonizing the host antiviral response. Previous studies have suggested the involvement of various DExD/H-box RNA helicase such as DDX58 (also known as RIG-I) in the initiation of innate immune responses [66, 67]. In this study, DDX58 (DExD/H-box helicase 58) was the upregulated gene of IR and DR pigs and are often seen in the affected pathways, thereby suggesting that DDX58 plays an essential role in initiating an antiviral response [68]. Another important gene that corresponds to this pathway was upregulated gene of DR pigs (RNASEL), a principal mediator of the IFN-inducible antiviral state that can determine the survival of animals infected with highly pathogenic viruses [69, 70]. Based on those findings, influenza A pathway is crucial for leucocyte-related traits.

## 5. Conclusions

We investigated the transcriptome of leucocyte variation in peripheral blood of pigs induced by poly I:C. The microarray analysis identified many DEGs that were involved in the immune defense responses of both the IR and DR pigs, although the two groups showed different immune responses after dsRNA stimulation. Overall, our study will enhance the understanding of the molecular basis for the antiviral capacity of pigs and potentially important genes for disease resistance breeding.

## Figures and Tables

**Table 1:** Known immune regulatory DEGs of IR and DR.

**DEGs Category**	**Probe-sets ID**	**Gene Symbol**	**Log2 FC**	**Reference**
IR-Upregulated	Ssc.16234.1.S1_at	TCN1	3.1	[43]
	Ssc.18927.1.S1_at	MS4A8B	2.6	[31]
	Ssc.9117.1.S1_at	S100A12	2.3	[44]
	Ssc.6369.1.A1_at	CSF1	2.2	[45]
IR-Downregulated	Ssc.26709.1.S1_at	GPR183	-2.0	[46]
DR-Upregulated	Ssc.12829.1.A1_at	TNFSF10	3.5	[47]
	Ssc.26005.1.S1_at	ZBP1	3.3	[48]
	Ssc.8261.1.A1_at	CYP2C9	3.2	[49]
DR-Downregulated	Ssc.16671.1.S1_at	TGFBI	-3.9	[50]

**Table 2:** Major functional annotation clusters in poly I:C-induced leucocyte responders based on fold change (FC) and (p < 0.01).

**GO name**	**Gene count**	**Fold change**	**Genes**	
			**Expressed in increased leucocyte responders**	**Expressed in decreased leucocyte responders**
Innate immunity	10	18.20	DDX58, SH2D1A, CFB, RSAD2, TLR4, S100A12, ISG20	DDX58, CFB, RSAD2, TLR4, OAS1, MX1, MX2, ISG20
Innate immune response	14	7.18	DDX58, SH2D1A, S100A8, S100A9, RSAD2, TLR4, TNK2, S100A12, ISG20	DDX58, PTK2B, TRIM26, RSAD2, TLR4, OAS1, MX2, ISG20, TEC
Immunity	10	10.76	DDX58, SH2D1A, CFB, RSAD2, TLR4, S100A12, ISG20	DDX58, CFB, RSAD2, TLR4, OAS1, MX1, MX2, ISG20
Negative regulation of viral genome replication	6	22.47	-	PLSCR1, ISG15, RSAD2, OAS1, MX1, ISG20
Defense response to virus	9	6.68	IFIT3, PLSCR1, IFIT2, ISG15, RSAD2, OAS1, MX1, MX2, ISG20	PLSCR1, ISG15, RSAD2, OAS1, MX1, ISG20
Antiviral defense	6	9.10	DDX58, RSAD2, ISG20	DDX58, RSAD2, OAS1, MX1, MX2, ISG20

**Table 3:** Pathway regulated by DEGs of IR and DR.

**GO accession**	**KEGG pathway**	**Gene count**	**Genes**
ssc05164	Influenza A	9	DDX58, RNASEL, TNFSF10, IRF7, RSAD2, TLR4, OAS1, STAT1, MX1
ssc05162	Measles	7	DDX58, TNFSF10, IRF7, TLR4, OAS1, STAT1, MX1
ssc05168	Herpes simplex infection	6	DDX58, RNASEL, IRF7, OAS1, STAT1, DAXX
ssc04623	Cytosolic DNA-sensing pathway	4	DDX58, IRF7, TREX1, ZBP1
ssc05160	Hepatitis C	5	DDX58, RNASEL, IRF7, OAS1, STAT1
ssc05161	Hepatitis B	5	DDX58, PTK2B, IRF7, TLR4, STAT1
ssc04062	Chemokine signaling pathway	5	PTK2B, CCL8, GNB4, PAK1, STAT1
ssc04060	Cytokine-cytokine receptor interaction	5	ACVR1B, TNFSF10, IL1RAP, CCL8, IL13RA1
ssc04622	RIG-I-like receptor signaling pathway	3	DDX58, ISG15, IRF7

**Table 4:** Differentially expressed genes in the affected pathways. Negative fold change values indicate downregulation in response to poly I:C stimulation.

**Fold change**		
**IR vs. DR**		
**Gene**	**IR**	**DR**
**DDX58**	2.63	3.99
**RNASEL**	-	2.11
**TNFSF10**	-	3.45
**IRF7**	1.92	2.99
**RSAD2**	2.04	5.30
**TLR4**	2.18	3.56
**OAS1**	-	3.02
**STAT1**	-	2.38
**DAXX**	2.10	2.66
**MX1**	-	2.85
**TREX1**	1.86	2.34
**ZBP1**	-	3.29
**ACVR1B**	2.95	3.09
**IL1RAP**	2.28	2.95
**CCL8**	-	-2.69
**PTK2B**	1.93	2.16
**GNB4**	-	2.50
**PAK1**	-	2.20
**IL13RA1**	-	2.17
**ISG15**	-	2.33

## Data Availability

All data are publicly available and consent for use of all data is available.

## Supplementary Materials

Supplementary Materials Supplementary File 1: list of the differentially expressed genes (DEGs) analyzed in qPCR and primers used for their amplification. Supplementary File 2: differential gene expression of IR and DR pigs. Supplementary File 3: comparative functional classification of differentially expressed genes (DEGs) in IR and DR pigs. Supplementary File 4: pathway in the DR pigs.
