# Supplementary material for: Transcriptional Profiling of Leucocyte Count Variation from Porcine Peripheral Blood Reveals Differential Gene Expression
Source: Biomed Res Int. 2018 Nov 18;2018:1496536. doi: 10.1155/2018/1496536 (PMC6276489; doi:10.1155/2018/1496536)
Supplement: Supplementary Materials — Supplementary File 1: list of the differentially expressed genes (DEGs) analyzed in qPCR and primers used for their amplification. Supplementary File 2: differential gene expression of IR and DR pigs. Supplementary File 3: comparative functional classification of differentially expressed genes (DEGs) in IR and DR pigs. Supplementary File 4: pathway in the DR pigs. [file 1496536.f1.pdf]

List of the differentially expressed genes (DEGs) analyzed in qPCR and p
The table reports the gene IDs, gene annotations and sequences of the p

Gene	Gene annotation	Primers
RSAD2	radical S-adenosyl methionine domain containing 2	Forward
		Reverse
S100A12	S100 calcium binding protein A12	Forward
		Reverse
S100A8	S100 calcium binding protein A8	Forward
		Reverse
S100A9	S100 calcium binding protein A9	Forward
		Reverse
SH2D1A	SH2 domain containing 1A	Forward
		Reverse
ISG20	interferon stimulated exonuclease gene 20	Forward
		Reverse
TNK2	tyrosine kinase non receptor 2	Forward
		Reverse
OAS1	2'-5'-oligoadenylate synthetase 1	Forward
		Reverse
PLSCR1	phospholipid scramblase 1	Forward
		Reverse
MX1	MX dynamin like GTPase 1	Forward
		Reverse
MX2	myxovirus (influenza virus) resistance 2	Forward
		Reverse
TRIM26	tripartite motif containing 26	Forward
		Reverse
STAT1	signal transducer and activator of transcription 1	Forward
		Reverse
TLR4	toll like receptor 4	Forward
		Reverse
ACTB	actin beta	Forward
		Reverse

primers used for their amplification

rimer sets used to analyze the expression level of 14 DEGs including the houseke

Sequences (5'-3')	Product length (bp)
TATCTGGACATTCTTGCCAT	172
CATTGAAGCGATTGATGACT	
AAGCAGCTGATCACCAAGGAA	169
GGATGTTGTCGTGGGCAGTTA	
TGCCATTAACTCCCTCATCGAA	161
TGATGTCCAGCTCTTTGAACCA	
AACATCTTCCACCAGTACTCGG	242
GTCTTGTGCATCTCCTCGTG	
GCTATTTGCTGAGGGACA	272
TCTTCTTCCTGTAGCACCT	
TCCCGCCCCTGACTTGACT	171
ATGCCTCTTGCCATGTTCCAC	
AGCACTACCCCAGAATGACGAC	270
ACCATGACATTGTAGACGTCCT	
CACAGGTCAAGGAAGCCATC	239
TTCCAGCTGTCTCCGAAT	
AGAGATAGAAATCCAAGCTCC	152
CTTTGAAATCTTGCCAACCAC	
TAGACCTTCCTGGCATCACC	225
CCAGATCCGGCTTCGTCA	
AGCTGATGCCTAAACCCC	161
ACCCATCTGCCAGTTTAGAGG	
CAGAGCTCATGCAGGATACCA	262
CTGTAGGTCACGCACTTCCA	
AAACATAAGGAGCTTGACAG	165
CTGATCGTTCTTTGCCACA	
TTCTACATCAAGTGCCCCTA	261
TCAAATTGCACAGTTCCTC	
GGACTTCGAGCAGGAGATGG	233
GCACCGTGTTGGCGTAGAGG	

eping gene used as reference for normalization.

DEGs of increase responders (IR) pigs.

Probesets ID	Gene symbol	Log2FC	P.Value
Ssc.12431.1.A1_at	MYO5B	4.094010063	0.0013816
Ssc.23801.1.S1_at	RETN	3.775808068	1.73E-05
Ssc.22354.1.A1_at	BATF3	3.583885491	6.33E-05
Ssc.16234.1.S1_at	TCN1	3.08672676	0.0013682
Ssc.24144.1.S1_at	ACVR1B	2.945451929	0.0191245
Ssc.27433.1.S1_at	TGM1	2.902550062	0.0025993
Ssc.5683.1.S1_a_at	TOR3A	2.847993101	0.0013934
Ssc.336.1.S1_at	USP18	2.753741214	0.0041337
Ssc.30887.1.S1_at	TNFAIP6	2.751127135	0.0062499
Ssc.6168.1.S1_at	ETV7	2.63629099	0.0034567
Ssc.21.1.S1_s_at	DDX58	2.633643978	0.0331971
SscAffx.1.1.S1_at	ISG20	2.626876738	0.0002799
Ssc.19643.1.A1_at	COL4A4	2.591341426	0.0012444
Ssc.18927.1.S1_at	MS4A8B	2.576071825	0.0021085
Ssc.5987.1.A1_at	RHPN2	2.565553957	0.0139589
Ssc.21339.1.S1_at	TRIM21	2.48112539	0.0005581
Ssc.26300.2.S1_at	UNC93B1	2.451645961	0.0003616
Ssc.25255.1.S1_at	ASRGL1	2.424264603	0.0047803
Ssc.3012.1.S1_at	UPP1	2.405025147	0.000328
Ssc.16250.1.S2_at	IL1RN	2.351002641	0.0002723
Ssc.13769.1.S1_at	LOC728320	2.30837594	0.0202183
Ssc.9117.1.S1_at	S100A12	2.29901906	0.0011094
Ssc.7864.1.A1_at	IL1RAP	2.283415664	0.0089265
Ssc.9364.1.S1_at	EDN1	2.276514639	0.0174868
Ssc.8997.1.A1_at	PLTP	2.265180047	0.0468628
Ssc.6433.1.S1_at	ZNFX1	2.26344443	0.0008873
Ssc.6369.1.A1_at	CSF1	2.244841341	0.000613
Ssc.14444.3.A1_a_at	ARG2	2.240699891	0.0002939
Ssc.646.1.S1_at	CSTA	2.193947487	0.0144002
Ssc.12781.1.A1_s_at	TLR4	2.181352466	0.0071606
Ssc.27370.1.S1_at	SLCO4C1	2.140740274	0.0025911
Ssc.3452.1.A1_at	DDX26B	2.112435761	0.0012773
Ssc.11239.1.A1_at	DNAJA1	2.106150702	0.0016209
Ssc.26626.1.S1_at	VCPIP1	2.102766943	0.0039332
Ssc.16903.1.S1_at	DAXX	2.102366882	0.0038088
Ssc.3706.1.S2_at	SOD2	2.089581753	0.0013668
Ssc.12446.1.A1_at	CASP4	2.073749114	0.0028251
Ssc.11255.1.A1_at	ACSL6	2.065705133	0.0020461
Ssc.21872.1.S1_at	LOC57228	2.051680872	0.0054061
Ssc.18272.1.A1_at	TCIRG1	2.048556574	0.0003484
AFFX-Ss_IRP_5_at	RSAD2	2.040688035	0.0020012
Ssc.2381.1.A1_at	S100A9	2.040081686	0.0029864
Ssc.6191.1.S1_at	DTX3L	2.038638755	0.0217514
Ssc.30833.1.S1_at	LOC728830	2.03542381	0.0454051
Ssc.2688.1.S1_at	MICALL2	2.027719219	0.0043606
Ssc.10460.1.S1_at	CFB	2.027045031	0.0087569
Ssc.3719.1.S1_at	TIFA	2.026447746	0.0394709
Ssc.17100.1.S1_at	S100A8	2.014394236	0.0065245

Ssc.5026.2.S1_at	NUDT3	2.010212094	0.0085915
Ssc.6718.1.S1_at	C15orf15	-2.005979087	0.0120398
Ssc.21670.1.S1_at	SYNCRIP	-2.010710824	0.003248
Ssc.6515.1.A1_at	PSPC1	-2.018842339	0.0005225
Ssc.29439.1.A1_at	GALNT7	-2.02168232	0.0018225
Ssc.1551.1.S1_at	PLXND1	-2.022289184	0.0020398
Ssc.18589.3.A1_at	SH3BGRL2	-2.033287292	0.0104612
Ssc.15404.1.S1_at	CENPH	-2.035861073	0.0143172
Ssc.8127.1.A1_at	DDX46	-2.036150464	0.0086573
Ssc.22527.1.A1_at	PBX3	-2.036349081	0.0004765
Ssc.2543.1.S1_at	SPOCK2	-2.03934937	0.0005484
Ssc.26709.1.S1_at	GPR183	-2.039891943	0.0109199
Ssc.2033.1.S1_at	CRY1	-2.04160133	0.006524
Ssc.19359.2.S1_at	MBP	-2.043375158	0.0060951
Ssc.10883.2.A1_at	TNRC6A	-2.045291132	0.0139418
Ssc.24353.1.S1_at	VRK1	-2.046572126	0.0059335
Ssc.1135.1.A1_at	PRKAR2B	-2.048245116	0.0244537
Ssc.29140.1.A1_at	MAN2A1	-2.049986602	0.0045617
Ssc.13482.1.S1_at	B3GALNT1	-2.050561769	0.0027616
Ssc.15291.1.A1_at	C5orf23	-2.064178244	0.0066005
Ssc.8658.1.A1_at	PRKACB	-2.067927491	0.004096
Ssc.1856.1.A1_at	ACAA2	-2.069284822	0.0009638
Ssc.5470.1.S1_at	OSBPL8	-2.070737939	0.0077977
Ssc.31017.1.A1_at	KIAA0776	-2.097463337	0.0040772
Ssc.6338.2.S1_at	USP1	-2.097827528	0.0238283
Ssc.30164.1.A1_at	PIP3-E	-2.102421636	0.0146302
Ssc.25162.1.S1_at	HNRNPA3	-2.102580581	0.0078847
Ssc.2595.1.S1_at	OSGEPL1	-2.102902109	0.0012683
Ssc.7694.1.A1_at	TOPBP1	-2.117791209	0.0026656
Ssc.2868.1.S1_at	PSIP1	-2.12244046	0.013915
Ssc.29750.1.A1_at	TP53INP1	-2.122464018	0.011099
Ssc.7804.1.A1_at	LYST	-2.126372419	0.0011373
Ssc.19482.1.A1_at	ATP8A1	-2.129651461	0.0046621
Ssc.4156.1.A1_at	OTUD6B	-2.129743758	0.0072188
Ssc.28142.1.A1_at	PTP4A1	-2.141055328	0.0027745
Ssc.20954.2.S1_a_at	MRPL3	-2.141289281	0.0035853
Ssc.30815.1.A1_at	SSB	-2.146225973	0.0096212
Ssc.10754.1.A1_at	PIK3R1	-2.157076969	0.0150847
Ssc.12561.1.A1_at	BCAT1	-2.161392271	0.0004083
Ssc.7818.1.A1_at	ABCB10	-2.168377391	0.0114959
Ssc.18849.1.A1_at	SH2D1A	-2.171514299	0.0263094
Ssc.7093.3.S1_at	HPSE	-2.173294466	0.0192243
Ssc.16489.1.S1_at	IL7R	-2.173602668	0.0039252
Ssc.7311.1.A1_at	TMEM161B	-2.178385257	0.0008736
Ssc.6670.2.S1_at	SLC39A6	-2.18144959	0.006876
Ssc.25221.1.A1_at	CDC14B	-2.182606787	0.0089044
Ssc.22557.1.A1_at	C4orf34	-2.186246645	0.006982
Ssc.4196.1.A1_at	ZBTB44	-2.193193834	0.0152256
Ssc.25265.1.S1_a_at	DHX29	-2.197702954	0.004036
Ssc.14379.1.A1_at	SLC38A1	-2.197910359	0.0004031

Ssc.16531.1.S1_at	AGPAT5	-2.20032609	0.0072683
Ssc.12654.1.A1_at	TNK2	-2.22714408	0.00517
Ssc.11661.2.S1_at	PPP1CC	-2.234263929	0.0087134
Ssc.8725.1.A1_at	BCL11B	-2.238316806	0.0018406
Ssc.8475.1.A1_at	CPSF6	-2.256213773	0.0020609
Ssc.14302.1.A1_at	APPL1	-2.264309487	0.0041221
Ssc.1429.1.A1_at	NAP1L1	-2.266165559	0.0008653
Ssc.7980.2.A1_at	ZNF22	-2.291982075	0.0021043
Ssc.6028.1.S1_at	OCC-1	-2.315256177	0.003458
Ssc.22060.1.A1_at	ZKSCAN1	-2.316335362	0.0009387
Ssc.3152.1.A1_at	MBTD1	-2.316402597	0.0010955
Ssc.11477.3.A1_at	IMMP2L	-2.335041117	0.004667
Ssc.4141.2.S1_at	KCTD12	-2.373766676	0.0001845
Ssc.4615.1.A1_at	MAP4K5	-2.379905093	0.0004649
Ssc.30959.1.A1_at	KIF7	-2.394170991	0.0026816
Ssc.14221.1.S1_at	NUDT21	-2.408870095	0.0021898
Ssc.23262.1.A1_s_at	CCDC50	-2.435005925	0.0027183
Ssc.7066.1.S1_at	MIB1	-2.435690543	0.002331
Ssc.2960.1.A1_at	ATP11A	-2.441666038	0.0006669
Ssc.8375.1.A1_at	RNF128	-2.497337801	0.0021142
Ssc.1139.1.A1_at	SLC4A4	-2.574216316	0.0018849
Ssc.6697.1.S1_at	SOX4	-2.646754763	0.0003341
Ssc.18388.1.A1_at	FAM105A	-2.662498084	0.0024997
Ssc.8442.1.A1_at	STK39	-2.673397235	0.0005608
Ssc.27084.1.S1_at	GPR37	-2.71556388	0.0201586
Ssc.7438.1.A1_at	SLC25A24	-2.717451213	0.0023049
Ssc.29623.1.A1_at	RNF144A	-2.827412196	7.14E-05
Ssc.6662.1.S1_at	SEMA6D	-2.835835716	0.0022459
Ssc.26573.1.S1_at	RALGPS2	-2.839473657	0.0005753
Ssc.19640.1.A1_at	FCER1A	-3.12968532	0.0008637
Ssc.21291.1.A1_at	LOC100129387	-3.328739351	1.06E-05

DEGs of decrease responder (DR) pigs

Probesets ID	Gene Symbol	Log2FC	P.Value
AFFX-Ss_IRP_3_at	RSAD2	5.304039196	4.38E-06
Ssc.16250.1.S1_at	IL1RN	4.607890743	2.10E-06
Ssc.23801.1.S1_at	RETN	4.189068671	2.59E-07
SscAffx.1.1.S1_at	ISG20	4.136246536	2.52E-05
Ssc.21.1.S1_s_at	DDX58	3.991869073	3.02E-05
Ssc.6168.1.S1_at	ETV7	3.88058293	5.30E-06
Ssc.9364.1.S1_at	EDN1	3.777566396	0.0002311
Ssc.29329.1.A1_at	DCHS2	3.612296848	3.52E-06
Ssc.10049.1.A1_at	HIATL1	3.612275169	2.72E-05
Ssc.16103.1.S1_s_at	CRISP2	3.585440168	2.49E-06
Ssc.12781.1.A1_at	TLR4	3.56217798	0.0003162
Ssc.336.1.S1_at	USP18	3.503408736	1.63E-06
Ssc.5145.2.S1_at	HSPA1A	3.50026051	0.0001242
Ssc.12918.1.S1_at	NMI	3.462458124	6.28E-06
Ssc.12431.1.A1_at	MYO5B	3.448634305	1.13E-06
Ssc.12829.1.A1_at	TNFSF10	3.446131328	2.13E-05
Ssc.22620.1.S1_at	IFIT2	3.342441365	0.0001696
Ssc.26005.1.S1_at	ZBP1	3.290758751	4.17E-05
Ssc.10460.1.S1_at	CFB	3.234609347	7.39E-06
Ssc.18948.1.S1_at	SAA1	3.220900679	4.83E-05
Ssc.8261.1.A1_at	CYP2C9	3.185045357	4.00E-05
Ssc.1024.1.S1_at	SNTB1	3.124389094	4.99E-06
Ssc.21339.1.S1_at	TRIM21	3.098573375	1.86E-06
Ssc.24144.1.S1_at	ACVR1B	3.09324303	2.90E-06
Ssc.28988.1.S1_at	CNP	3.048814653	1.48E-05
Ssc.220.1.A1_at	MX2	3.048585713	3.92E-06
Ssc.5683.1.S1_a_at	TOR3A	3.031097096	3.28E-06
Ssc.1031.1.S1_at	OAS1	3.02325665	0.0014731
Ssc.3719.1.S1_at	TIFA	3.022718635	8.19E-06
Ssc.6433.2.S1_at	ZNFX1	3.009727117	2.87E-05
Ssc.26300.2.S1_at	UNC93B1	3.001128127	1.06E-05
Ssc.25739.1.S1_at	IRF7	2.990352599	0.0002388
Ssc.25255.2.S1_a_at	ASRGL1	2.969042504	1.48E-05
Ssc.7864.1.A1_at	IL1RAP	2.950173737	5.50E-05
Ssc.3706.1.S1_at	SOD2	2.917184637	0.0001097
Ssc.6191.1.S1_at	DTX3L	2.893128585	5.00E-05
Ssc.17458.1.S1_at	DMXL2	2.892959645	1.56E-05
Ssc.8473.1.S2_at	DNAJA1	2.869401235	2.01E-05
Ssc.20294.1.S1_at	LOC441108	2.856935225	0.0001261
Ssc.221.1.S1_at	MX1	2.84932932	0.0001138
Ssc.2688.1.S1_at	MICALL2	2.79466907	7.81E-06
Ssc.22354.1.A1_at	BATF3	2.789114722	1.95E-05
Ssc.3773.1.S1_at	CASP7	2.787177913	7.09E-05
Ssc.30869.1.S1_at	BAT2D1	2.787034965	6.34E-06
Ssc.12446.1.A1_at	CASP4	2.780595846	5.37E-05
Ssc.19763.1.S1_at	MED25	2.777765326	1.35E-05
Ssc.27433.1.S1_at	TGM1	2.702362685	2.50E-05

Ssc.16903.1.S1_at	DAXX	2.658093543	2.11E-05
Ssc.23929.1.A1_at	PLSCR1	2.649541185	0.0002822
Ssc.12756.1.A1_at	RHBDD1	2.625583586	5.79E-05
Ssc.5987.1.A1_at	RHPN2	2.622995058	0.0001273
Ssc.2101.1.S1_at	FTSJD2	2.580575907	1.10E-05
Ssc.1217.1.S1_at	PHF11	2.55770657	5.99E-05
Ssc.5743.2.A1_at	HK3	2.555131292	7.74E-05
Ssc.18272.1.A1_at	TCIRG1	2.532974141	3.93E-05
Ssc.2572.1.S1_at	PPA1	2.520763901	4.63E-05
Ssc.28913.1.A1_at	GNB4	2.500590752	0.0001373
Ssc.30887.1.S1_at	TNFAIP6	2.500090028	0.0004409
Ssc.17902.1.A1_at	TDRD7	2.496948075	8.25E-05
Ssc.31140.1.S1_at	IFIT3	2.487025366	0.000551
Ssc.5026.2.S1_at	NUDT3	2.462542251	6.35E-05
Ssc.26626.1.S1_at	VCPIP1	2.44946814	0.000478
Ssc.6371.1.A1_at	LOC100128809	2.434853412	0.0009363
Ssc.61.1.S1_at	LOC100133511	2.432919412	0.0001133
Ssc.12191.1.A1_at	HSP90AA1	2.429984935	0.0011778
Ssc.6025.1.S1_at	STAT1	2.382102727	0.0002681
Ssc.7812.1.A1_at	STK3	2.375086455	0.0002588
Ssc.30724.1.S1_at	HERC6	2.3708887	5.60E-05
Ssc.20062.1.A1_at	TRAFD1	2.343454831	8.65E-05
Ssc.1143.3.S1_a_at	ANXA4	2.342817772	0.0009567
Ssc.12795.1.S1_at	TREX1	2.338074527	4.80E-05
Ssc.851.1.A1_at	ETV6	2.328742522	9.14E-05
Ssc.11557.1.A1_at	ISG15	2.328164413	0.0001577
Ssc.14244.1.A1_at	UBR1	2.313542395	4.10E-05
Ssc.29054.2.S1_at	GBP3	2.24764538	0.000295
Ssc.20734.1.S1_at	TRIM26	2.24699274	6.53E-05
Ssc.5228.1.S1_at	LGALS8	2.23855834	0.0010148
Ssc.7224.1.A1_at	LBA1	2.237173582	0.0005455
Ssc.9733.1.S1_at	MORC3	2.234646715	0.0001624
Ssc.3214.2.S1_at	RNASET2	2.228646277	0.0001694
Ssc.4466.1.S1_at	TEC	2.22678257	6.88E-05
Ssc.646.1.S1_at	CSTA	2.224037011	7.38E-05
Ssc.10593.2.A1_at	IFI44L	2.221457918	0.0007915
Ssc.18586.1.A1_at	MAD2L2	2.214654584	9.68E-05
Ssc.29301.1.A1_at	C19orf66	2.200862762	8.63E-05
Ssc.24652.1.S1_at	PAK1	2.200493001	0.0005028
Ssc.13226.1.A1_at	PARP9	2.19461663	0.0001456
Ssc.864.1.S1_at	CFH	2.191332109	0.000781
Ssc.3012.1.S1_at	UPP1	2.187162871	0.0007884
Ssc.17245.1.S1_at	IL13RA1	2.169024933	0.0010022
Ssc.6906.1.A1_at	FNDC3A	2.164539785	8.74E-05
Ssc.2140.1.S1_at	PTK2B	2.156304441	0.0001676
Ssc.15306.1.S1_at	GGH	2.150927794	0.000444
Ssc.15720.1.A1_at	CNDP2	2.150044034	0.0002102
Ssc.5685.1.S1_at	ANKFY1	2.149556871	6.43E-05
Ssc.7274.1.A1_at	EIF2AK2	2.147600506	0.0001628
Ssc.28568.1.A1_at	PDE12	2.128312555	0.0004581

Ssc.18805.1.A1_at	RNASEL	2.118799269	0.0002826
Ssc.9766.3.A1_a_at	C21orf66	2.107058612	0.0005327
Ssc.5119.1.S1_at	SLC25A28	2.090947508	0.0002869
Ssc.11079.1.A1_at	RNASE4	2.089285582	0.0020863
Ssc.27370.1.S1_at	SLCO4C1	2.083427522	0.0010761
Ssc.12286.2.A1_at	PML	2.082013935	0.0001604
Ssc.1755.2.A1_at	NANS	2.079042436	8.78E-05
Ssc.4539.1.S1_at	WHSC1L1	2.076160546	9.55E-05
Ssc.30665.1.S1_at	C4orf32	2.059887921	0.0024497
Ssc.27135.1.A1_at	FOXS1	2.037541071	0.0001171
Ssc.26216.2.A1_at	SOCS1	2.033052315	0.0003359
Ssc.6172.1.S1_at	XAF1	2.033018111	0.0006778
Ssc.607.1.S1_at	SLC2A3	2.019934252	0.0003884
Ssc.7914.1.S1_at	USP25	2.013635109	0.000133
Ssc.26085.1.S1_at	STARD8	2.006793086	0.000136
Ssc.114.1.S1_at	HSPA6	2.004611159	0.0009547
Ssc.18929.1.S1_s_at	RNF128	-2.003792106	0.0105651
Ssc.18897.1.S1_at	FAM135A	-2.034994174	0.0035609
Ssc.29623.1.A1_at	RNF144A	-2.064918029	0.0136457
Ssc.1551.1.S1_at	PLXND1	-2.070062125	0.0017693
Ssc.7804.1.A1_at	LYST	-2.168759659	0.0053664
Ssc.25324.1.S1_at	DMD	-2.172729073	0.0050767
Ssc.17560.1.S1_at	GPR177	-2.21419904	0.0003118
Ssc.9471.1.A1_at	FAM108C1	-2.232825136	0.0071804
Ssc.15640.1.S1_at	MT2A	-2.24214614	0.0002325
Ssc.14467.2.S1_a_at	AREGB	-2.345459599	0.0001729
Ssc.5453.1.A1_at	QPCT	-2.345676288	0.000809
Ssc.14361.1.A1_at	OLFM1	-2.360937863	0.0007601
Ssc.15291.1.A1_at	C5orf23	-2.4029174	0.0004558
Ssc.18388.1.A1_at	FAM105A	-2.423988618	5.14E-05
Ssc.6974.1.A1_at	SYTL2	-2.436128846	0.0012614
Ssc.4103.1.A1_at	FNDC3B	-2.471305808	0.0005948
Ssc.28522.1.S1_at	SEPN1	-2.524221234	0.0003246
Ssc.1320.1.A1_at	LGALS1	-2.556338238	5.31E-05
Ssc.420.4.S1_a_at	CAMP	-2.635386044	0.0003765
Ssc.9957.1.A1_at	CCL8	-2.699671486	0.002129
Ssc.8767.1.A1_at	CYP1B1	-3.273345691	7.32E-06
Ssc.31189.1.S1_at	CHPT1	-3.786627776	3.02E-06
Ssc.16671.1.S1_at	TGFBI	-3.907514613	0.0006281

DEGs specific to IR pigs

Probesets ID	Gene Symbol	Log2FC	P.Value
Ssc.16234.1.S1_at	TCN1	3.086727	0.001368227
Ssc.19643.1.A1_at	COL4A4	2.591341	0.001244406
Ssc.18927.1.S1_at	MS4A8B	2.576072	0.002108541
Ssc.13769.1.S1_at	LOC728320	2.308376	0.020218299
Ssc.9117.1.S1_at	S100A12	2.299019	0.001109425
Ssc.8997.1.A1_at	PLTP	2.26518	0.046862824
Ssc.6369.1.A1_at	CSF1	2.244841	0.000612965
Ssc.14444.3.A1_a_at	ARG2	2.2407	0.000293894
Ssc.3452.1.A1_at	DDX26B	2.112436	0.001277341
Ssc.11255.1.A1_at	ACSL6	2.065705	0.002046089
Ssc.21872.1.S1_at	LOC57228	2.051681	0.005406147
Ssc.2381.1.A1_at	S100A9	2.040082	0.002986434
Ssc.30833.1.S1_at	LOC728830	2.035424	0.045405056
Ssc.17100.1.S1_at	S100A8	2.014394	0.006524463
Ssc.6718.1.S1_at	C15orf15	-2.00598	0.012039775
Ssc.21670.1.S1_at	SYNCRIP	-2.01071	0.00324796
Ssc.6515.1.A1_at	PSPC1	-2.01884	0.000522464
Ssc.29439.1.A1_at	GALNT7	-2.02168	0.001822546
Ssc.18589.3.A1_at	SH3BGRL2	-2.03329	0.010461162
Ssc.15404.1.S1_at	CENPH	-2.03586	0.014317222
Ssc.8127.1.A1_at	DDX46	-2.03615	0.008657343
Ssc.22527.1.A1_at	PBX3	-2.03635	0.000476487
Ssc.2543.1.S1_at	SPOCK2	-2.03935	0.000548417
Ssc.26709.1.S1_at	GPR183	-2.03989	0.010919886
Ssc.2033.1.S1_at	CRY1	-2.0416	0.006524039
Ssc.19359.2.S1_at	MBP	-2.04338	0.006095099
Ssc.10883.2.A1_at	TNRC6A	-2.04529	0.013941795
Ssc.24353.1.S1_at	VRK1	-2.04657	0.005933548
Ssc.1135.1.A1_at	PRKAR2B	-2.04825	0.024453749
Ssc.29140.1.A1_at	MAN2A1	-2.04999	0.004561666
Ssc.13482.1.S1_at	B3GALNT1	-2.05056	0.002761565
Ssc.8658.1.A1_at	PRKACB	-2.06793	0.004095972
Ssc.1856.1.A1_at	ACAA2	-2.06928	0.000963763
Ssc.5470.1.S1_at	OSBPL8	-2.07074	0.007797738
Ssc.31017.1.A1_at	KIAA0776	-2.09746	0.00407719
Ssc.6338.2.S1_at	USP1	-2.09783	0.023828316
Ssc.30164.1.A1_at	PIP3-E	-2.10242	0.014630199
Ssc.25162.1.S1_at	HNRNPA3	-2.10258	0.0078847
Ssc.2595.1.S1_at	OSGEPL1	-2.1029	0.001268252
Ssc.7694.1.A1_at	TOPBP1	-2.11779	0.002665552
Ssc.2868.1.S1_at	PSIP1	-2.12244	0.013914951
Ssc.29750.1.A1_at	TP53INP1	-2.12246	0.011098953
Ssc.19482.1.A1_at	ATP8A1	-2.12965	0.00466214
Ssc.4156.1.A1_at	OTUD6B	-2.12974	0.007218834
Ssc.28142.1.A1_at	PTP4A1	-2.14106	0.002774463
Ssc.20954.2.S1_a_at	MRPL3	-2.14129	0.003585323
Ssc.30815.1.A1_at	SSB	-2.14623	0.009621241

Ssc.10754.1.A1_at	PIK3R1	-2.15708	0.015084659
Ssc.12561.1.A1_at	BCAT1	-2.16139	0.00040826
Ssc.7818.1.A1_at	ABCB10	-2.16838	0.011495856
Ssc.18849.1.A1_at	SH2D1A	-2.17151	0.026309448
Ssc.7093.3.S1_at	HPSE	-2.17329	0.019224287
Ssc.16489.1.S1_at	IL7R	-2.1736	0.003925216
Ssc.7311.1.A1_at	TMEM161B	-2.17839	0.000873618
Ssc.6670.2.S1_at	SLC39A6	-2.18145	0.006875997
Ssc.25221.1.A1_at	CDC14B	-2.18261	0.008904415
Ssc.22557.1.A1_at	C4orf34	-2.18625	0.006982027
Ssc.4196.1.A1_at	ZBTB44	-2.19319	0.01522565
Ssc.25265.1.S1_a_at	DHX29	-2.1977	0.004035962
Ssc.14379.1.A1_at	SLC38A1	-2.19791	0.000403112
Ssc.16531.1.S1_at	AGPAT5	-2.20033	0.007268285
Ssc.12654.1.A1_at	TNK2	-2.22714	0.005170009
Ssc.11661.2.S1_at	PPP1CC	-2.23426	0.008713381
Ssc.8725.1.A1_at	BCL11B	-2.23832	0.001840612
Ssc.8475.1.A1_at	CPSF6	-2.25621	0.002060896
Ssc.14302.1.A1_at	APPL1	-2.26431	0.004122061
Ssc.1429.1.A1_at	NAP1L1	-2.26617	0.000865253
Ssc.7980.2.A1_at	ZNF22	-2.29198	0.002104305
Ssc.6028.1.S1_at	OCC-1	-2.31526	0.003458
Ssc.22060.1.A1_at	ZKSCAN1	-2.31634	0.000938701
Ssc.3152.1.A1_at	MBTD1	-2.3164	0.001095543
Ssc.11477.3.A1_at	IMMP2L	-2.33504	0.004666992
Ssc.4141.2.S1_at	KCTD12	-2.37377	0.00018453
Ssc.4615.1.A1_at	MAP4K5	-2.37991	0.000464922
Ssc.30959.1.A1_at	KIF7	-2.39417	0.002681566
Ssc.14221.1.S1_at	NUDT21	-2.40887	0.002189772
Ssc.23262.1.A1_s_at	CCDC50	-2.43501	0.00271826
Ssc.7066.1.S1_at	MIB1	-2.43569	0.002331006
Ssc.2960.1.A1_at	ATP11A	-2.44167	0.000666856
Ssc.1139.1.A1_at	SLC4A4	-2.57422	0.001884852
Ssc.6697.1.S1_at	SOX4	-2.64675	0.0003341
Ssc.8442.1.A1_at	STK39	-2.6734	0.000560791
Ssc.27084.1.S1_at	GPR37	-2.71556	0.020158616
Ssc.7438.1.A1_at	SLC25A24	-2.71745	0.002304912
Ssc.6662.1.S1_at	SEMA6D	-2.83584	0.002245909
Ssc.26573.1.S1_at	RALGPS2	-2.83947	0.000575254
Ssc.19640.1.A1_at	FCER1A	-3.12969	0.000863742
Ssc.21291.1.A1_at	LOC100129387	-3.32874	1.05948E-05

DEGs specific to DR pigs

Probesets ID	Gene Symbol	Log2FC	P.Value
Ssc.29329.1.A1_at	DCHS2	3.612296848	0.00000352
Ssc.10049.1.A1_at	HIATL1	3.612275169	0.0000272
Ssc.16103.1.S1_s_at	CRISP2	3.585440168	0.00000249
Ssc.5145.2.S1_at	HSPA1A	3.50026051	0.000124206
Ssc.12918.1.S1_at	NMI	3.462458124	0.00000628
Ssc.12829.1.A1_at	TNFSF10	3.446131328	0.0000213
Ssc.22620.1.S1_at	IFIT2	3.342441365	0.000169642
Ssc.26005.1.S1_at	ZBP1	3.290758751	0.0000417
Ssc.18948.1.S1_at	SAA1	3.220900679	0.0000483
Ssc.8261.1.A1_at	CYP2C9	3.185045357	0.00004
Ssc.1024.1.S1_at	SNTB1	3.124389094	0.00000499
Ssc.28988.1.S1_at	CNP	3.048814653	0.0000148
Ssc.220.1.A1_at	MX2	3.048585713	0.00000392
Ssc.1031.1.S1_at	OAS1	3.02325665	0.001473091
Ssc.25739.1.S1_at	IRF7	2.990352599	0.000238757
Ssc.17458.1.S1_at	DMXL2	2.892959645	0.0000156
Ssc.20294.1.S1_at	LOC441108	2.856935225	0.000126126
Ssc.221.1.S1_at	MX1	2.84932932	0.000113757
Ssc.3773.1.S1_at	CASP7	2.787177913	0.0000709
Ssc.30869.1.S1_at	BAT2D1	2.787034965	0.00000634
Ssc.19763.1.S1_at	MED25	2.777765326	0.0000135
Ssc.23929.1.A1_at	PLSCR1	2.649541185	0.000282237
Ssc.12756.1.A1_at	RHBDD1	2.625583586	0.0000579
Ssc.2101.1.S1_at	FTSJD2	2.580575907	0.000011
Ssc.1217.1.S1_at	PHF11	2.55770657	0.0000599
Ssc.5743.2.A1_at	HK3	2.555131292	0.0000774
Ssc.2572.1.S1_at	PPA1	2.520763901	0.0000463
Ssc.28913.1.A1_at	GNB4	2.500590752	0.000137313
Ssc.17902.1.A1_at	TDRD7	2.496948075	0.0000825
Ssc.31140.1.S1_at	IFIT3	2.487025366	0.000551044
Ssc.6371.1.A1_at	LOC100128809	2.434853412	0.000936319
Ssc.61.1.S1_at	LOC100133511	2.432919412	0.000113282
Ssc.12191.1.A1_at	HSP90AA1	2.429984935	0.00117776
Ssc.6025.1.S1_at	STAT1	2.382102727	0.000268057
Ssc.7812.1.A1_at	STK3	2.375086455	0.000258799
Ssc.30724.1.S1_at	HERC6	2.3708887	0.000056
Ssc.20062.1.A1_at	TRAFD1	2.343454831	0.0000865
Ssc.1143.3.S1_a_at	ANXA4	2.342817772	0.000956712
Ssc.12795.1.S1_at	TREX1	2.338074527	0.000048
Ssc.851.1.A1_at	ETV6	2.328742522	0.0000914
Ssc.11557.1.A1_at	ISG15	2.328164413	0.000157715
Ssc.14244.1.A1_at	UBR1	2.313542395	0.000041
Ssc.29054.2.S1_at	GBP3	2.24764538	0.000294965
Ssc.20734.1.S1_at	TRIM26	2.24699274	0.0000653

Ssc.5228.1.S1_at	LGALS8	2.23855834	0.001014778
Ssc.7224.1.A1_at	LBA1	2.237173582	0.0005455
Ssc.9733.1.S1_at	MORC3	2.234646715	0.000162423
Ssc.3214.2.S1_at	RNASET2	2.228646277	0.000169358
Ssc.4466.1.S1_at	TEC	2.22678257	0.0000688
Ssc.10593.2.A1_at	IFI44L	2.221457918	0.000791496
Ssc.18586.1.A1_at	MAD2L2	2.214654584	0.0000968
Ssc.29301.1.A1_at	C19orf66	2.200862762	0.0000863
Ssc.24652.1.S1_at	PAK1	2.200493001	0.000502759
Ssc.13226.1.A1_at	PARP9	2.19461663	0.00014557
Ssc.864.1.S1_at	CFH	2.191332109	0.00078102
Ssc.17245.1.S1_at	IL13RA1	2.169024933	0.001002163
Ssc.6906.1.A1_at	FNDC3A	2.164539785	0.0000874
Ssc.2140.1.S1_at	PTK2B	2.156304441	0.000167589
Ssc.15306.1.S1_at	GGH	2.150927794	0.000444033
Ssc.15720.1.A1_at	CNDP2	2.150044034	0.00021024
Ssc.5685.1.S1_at	ANKFY1	2.149556871	0.0000643
Ssc.7274.1.A1_at	EIF2AK2	2.147600506	0.000162774
Ssc.28568.1.A1_at	PDE12	2.128312555	0.000458148
Ssc.18805.1.A1_at	RNASEL	2.118799269	0.000282558
Ssc.9766.3.A1_a_at	C21orf66	2.107058612	0.00053266
Ssc.5119.1.S1_at	SLC25A28	2.090947508	0.000286903
Ssc.11079.1.A1_at	RNASE4	2.089285582	0.002086332
Ssc.12286.2.A1_at	PML	2.082013935	0.000160413
Ssc.1755.2.A1_at	NANS	2.079042436	0.0000878
Ssc.4539.1.S1_at	WHSC1L1	2.076160546	0.0000955
Ssc.30665.1.S1_at	C4orf32	2.059887921	0.00244974
Ssc.27135.1.A1_at	FOXS1	2.037541071	0.000117062
Ssc.26216.2.A1_at	SOCS1	2.033052315	0.000335924
Ssc.6172.1.S1_at	XAF1	2.033018111	0.000677779
Ssc.607.1.S1_at	SLC2A3	2.019934252	0.000388423
Ssc.7914.1.S1_at	USP25	2.013635109	0.000133014
Ssc.26085.1.S1_at	STARD8	2.006793086	0.000135999
Ssc.114.1.S1_at	HSPA6	2.004611159	0.0009547
Ssc.18897.1.S1_at	FAM135A	-2.03499417	0.003560866
Ssc.25324.1.S1_at	DMD	-2.17272907	0.005076748
Ssc.17560.1.S1_at	GPR177	-2.21419904	0.000311829
Ssc.9471.1.A1_at	FAM108C1	-2.23282514	0.007180429
Ssc.15640.1.S1_at	MT2A	-2.24214614	0.000232527
Ssc.14467.2.S1_a_at	AREGB	-2.3454596	0.000172886
Ssc.5453.1.A1_at	QPCT	-2.34567629	0.000809025
Ssc.14361.1.A1_at	OLFM1	-2.36093786	0.000760094
Ssc.6974.1.A1_at	SYTL2	-2.43612885	0.001261386
Ssc.4103.1.A1_at	FNDC3B	-2.47130581	0.000594768
Ssc.28522.1.S1_at	SEPN1	-2.52422123	0.00032463
Ssc.1320.1.A1_at	LGALS1	-2.55633824	0.0000531
Ssc.420.4.S1_a_at	CAMP	-2.63538604	0.000376522

Ssc.9957.1.A1_at	CCL8	-2.69967149	0.00212902
Ssc.8767.1.A1_at	CYP1B1	-3.27334569	0.00000732
Ssc.31189.1.S1_at	CHPT1	-3.78662778	0.00000302
Ssc.16671.1.S1_at	TGFBI	-3.90751461	0.00062813

Common DEGs between IR and DR pigs

35 common elements in "IR Vs DR upregulated genes"	6 commor
MYO5B	PLXND1
RETN	C5orf23
BATF3	LYST
ACVR1B	RNF128
TGM1	FAM105A
TOR3A	RNF144A
USP18	
TNFAIP6	
ETV7	
DDX58	
ISG20	
RHPN2	
TRIM21	
UNC93B1	
ASRGL1	
UPP1	
IL1RN	
IL1RAP	
EDN1	
ZNFX1	
CSTA	
TLR4	
SLCO4C1	
DNAJA1	
VCPIP1	
DAXX	
SOD2	
CASP4	
TCIRG1	
RSAD2	
DTX3L	
MICALL2	
CFB	
TIFA	
NUDT3	

n elements in "IR Vs DR Downregulated genes"

Comparative (functional classification) of differentially ex

	IR		
**Term_IR**	**Count_IR**	**%_IR**	**Pvalue_IR**
GO:0045087~innate immune response*	13	11.40351	4.39622E-06
GO:0006955~immune response*	16	14.03509	0.000141159
GO:0051049~regulation of transport*	18	15.78947	0.00027454
GO:0031349~positive regulation of defense	7	6.140351	0.000763953
GO:0080134~regulation of response to stre	13	11.40351	0.002728222
GO:0051046~regulation of secretion*	9	7.894737	0.004426807
GO:0006952~defense response*	14	12.2807	0.004816061
GO:1903321~negative regulation of protein	4	3.508772	0.005162758
GO:0048584~positive regulation of respons	16	14.03509	0.007398484
GO:0031396~regulation of protein ubiquitin	5	4.385965	0.009117438
GO:0009967~positive regulation of signal tr	13	11.40351	0.009413673
GO:0002252~immune effector process*	9	7.894737	0.009951344
GO:0002684~positive regulation of immune	9	7.894737	0.013011642
GO:0010647~positive regulation of cell com	13	11.40351	0.016000491
GO:0023056~positive regulation of signalin	13	11.40351	0.016713717
GO:0070647~protein modification by small	9	7.894737	0.020895675
GO:0001817~regulation of cytokine produc	7	6.140351	0.023481971
GO:0050776~regulation of immune respon	7	6.140351	0.030043982
GO:0001816~cytokine production*	7	6.140351	0.032475304
GO:1903530~regulation of secretion by cel	7	6.140351	0.032892685
GO:0045089~positive regulation of innate i	4	3.508772	0.033236985
GO:0031347~regulation of defense respons	7	6.140351	0.033313564
GO:0050778~positive regulation of immune	6	5.263158	0.0357072
GO:0009605~response to external stimulus	15	13.15789	0.036916498
GO:0046903~secretion*	9	7.894737	0.038929275
GO:0043506~regulation of JUN kinase activ	3	2.631579	0.042808283
GO:0030522~intracellular receptor signalin	4	3.508772	0.043632142
GO:1903901~negative regulation of viral lif	3	2.631579	0.049912503
GO:0070887~cellular response to chemical	16	14.03509	0.052332138
GO:0042089~cytokine biosynthetic process	3	2.631579	0.057408925
GO:0030097~hemopoiesis*	8	7.017544	0.058223077
GO:0042107~cytokine metabolic process*	3	2.631579	0.059340994
GO:0080135~regulation of cellular respons	6	5.263158	0.059836741
GO:0045088~regulation of innate immune	4	3.508772	0.060103613
GO:0051223~regulation of protein transpor	7	6.140351	0.063953798
GO:0048525~negative regulation of viral pr	3	2.631579	0.065269473
GO:0009966~regulation of signal transduct	17	14.91228	0.065357997
GO:0060429~epithelium development*	9	7.894737	0.073942418
GO:0048534~hematopoietic or lymphoid o	8	7.017544	0.076787997
GO:1902533~positive regulation of intracel	8	7.017544	0.078689887
GO:0030518~intracellular steroid hormone	3	2.631579	0.079821647
GO:0002221~pattern recognition receptor	3	2.631579	0.08415012
GO:0002758~innate immune response-acti	3	2.631579	0.088548912
GO:0070201~regulation of establishment o	7	6.140351	0.090098046
GO:0019058~viral life cycle*	4	3.508772	0.091552217
GO:0002520~immune system development	8	7.017544	0.094189229

GO:0002218~activation of innate immune ı	3	2.631579	0.097545876

GO:0051050~positive regulation of transpo	11	9.649123	0.002152699
GO:0051047~positive regulation of secretic	6	5.263158	0.009544619
GO:0002449~lymphocyte mediated immun	5	4.385965	0.010283401
GO:0022008~neurogenesis	13	11.40351	0.012543346
GO:0070488~neutrophil aggregation	2	1.754386	0.014593084
GO:0002706~regulation of lymphocyte me	4	3.508772	0.015131827
GO:0009628~response to abiotic stimulus	9	7.894737	0.01592055
GO:0036211~protein modification process	23	20.17544	0.019616836
GO:0006464~cellular protein modification	23	20.17544	0.019616836
GO:0040011~locomotion	13	11.40351	0.019647203
GO:0007585~respiratory gaseous exchange	3	2.631579	0.020178415
GO:0090108~positive regulation of high-de	1	1.754386	0.021810551
GO:0002443~leukocyte mediated immunity	5	4.385965	0.025224327
GO:0071705~nitrogen compound transport	7	6.140351	0.028874783
GO:0090107~regulation of high-density lip	1	1.754386	0.028975802
GO:0002793~positive regulation of peptide	3	2.631579	0.02989537
GO:0051240~positive regulation of multice	12	10.52632	0.030377294
GO:0002703~regulation of leukocyte medi	4	3.508772	0.030656924
GO:0034599~cellular response to oxidative	4	3.508772	0.035927034

GO:0002523~leukocyte migration involved	2	1.754386	0.03608921
GO:0009408~response to heat	3	2.631579	0.036125655
GO:0031397~negative regulation of protei	3	2.631579	0.03775513
GO:0046903~secretion*	9	7.894737	0.038929275
GO:0008361~regulation of cell size	4	3.508772	0.040652784
GO:0006979~response to oxidative stress	5	4.385965	0.040735357
GO:0048699~generation of neurons	11	9.649123	0.040789213
GO:0048870~cell motility	11	9.649123	0.041432083
GO:0051674~localization of cell	11	9.649123	0.041432083
GO:0001909~leukocyte mediated cytotoxic	3	2.631579	0.042808283
GO:0032725~positive regulation of granulo	2	1.754386	0.043151147
GO:0021782~glial cell development	3	2.631579	0.044545985
GO:0002790~peptide secretion	4	3.508772	0.047770316
GO:0002708~positive regulation of lympho	3	2.631579	0.048098554
GO:0043588~skin development	4	3.508772	0.050996667
GO:0070486~leukocyte aggregation	6	5.263158	0.052274372
GO:0010942~positive regulation of cell dea	6	5.263158	0.056325222
GO:0015833~peptide transport	4	3.508772	0.056603771
GO:0033554~cellular response to stress	13	11.40351	0.056853797
GO:0018119~peptidyl-cysteine S-nitrosylat	2	1.754386	0.057122074
GO:0017014~protein nitrosylation	2	1.754386	0.057122074
GO:0032604~granulocyte macrophage colo	2	1.754386	0.057122074
GO:0032645~regulation of granulocyte ma	2	1.754386	0.057122074
GO:0007399~nervous system development	14	12.2807	0.057777374
GO:0006885~regulation of pH	3	2.631579	0.061295388
GO:0001819~positive regulation of cytokin	5	4.385965	0.061540302
GO:0030217~T cell differentiation	4	3.508772	0.062492394
GO:0032368~regulation of lipid transport	3	2.631579	0.063271687
GO:0002705~positive regulation of leukocy	3	2.631579	0.063271687
GO:0050729~positive regulation of inflamn	3	2.631579	0.065269473
GO:0042886~amide transport	4	3.508772	0.066157708
GO:0009314~response to radiation	5	4.385965	0.067012275
GO:0042752~regulation of circadian rhythn	3	2.631579	0.067288336
GO:0034380~high-density lipoprotein parti	2	1.754386	0.070891494
GO:0001906~cell killing	3	2.631579	0.071387666
GO:0007159~leukocyte cell-cell adhesion	6	5.263158	0.071901283
GO:0008544~epidermis development	4	3.508772	0.07248147
GO:0002697~regulation of immune effecto	5	4.385965	0.073707289
GO:0060429~epithelium development*	9	7.894737	0.073942418
GO:0030098~lymphocyte differentiation	5	4.385965	0.074690758
GO:0009266~response to temperature stin	3	2.631579	0.075566478
GO:0010875~positive regulation of cholest	2	1.754386	0.077701537
GO:0009416~response to light stimulus	4	3.508772	0.084517728
GO:0006928~movement of cell or subcellul	12	10.52632	0.084693947
GO:0065003~macromolecular complex ass	11	9.649123	0.085095593
GO:0043153~entrainment of circadian cloc	2	1.754386	0.091174066
GO:0032376~positive regulation of cholest	2	1.754386	0.091174066
GO:0032373~positive regulation of sterol tı	2	1.754386	0.091174066
GO:0009648~photoperiodism	2	1.754386	0.091174066
GO:0006869~lipid transport	4	3.508772	0.091552217

GO:0006935~chemotaxis	6	5.263158	0.092330835
GO:0045321~leukocyte activation	7	6.140351	0.092442995
GO:0042330~taxis	6	5.263158	0.093241595
GO:0055067~monovalent inorganic cation	3	2.631579	0.097545876
GO:0010874~regulation of cholesterol efflu	2	1.754386	0.097837254
GO:0030194~positive regulation of blood c	2	1.754386	0.097837254
GO:1900048~positive regulation of hemost	2	1.754386	0.097837254
GO:0010837~regulation of keratinocyte pro	2	1.754386	0.097837254
GO:0050820~positive regulation of coagula	2	1.754386	0.097837254
GO:0032101~regulation of response to exte	7	6.140351	0.0996771

(pressed genes (DEGs) involved in the biological process (BP) of IR

Genes_IR	Term_DR
S100A8, CFB, S100A9, SYNCRIP, RSAD2, TLR4	GO:0045087~innate immune response*
FCER1A, GPR183, S100A8, CFB, S100A9, SYN	GO:0006955~immune response*
MICALL2, FCER1A, ACAA2, S100A8, S100A9,	GO:0051049~regulation of transport*
DDX58, FCER1A, SH2D1A, S100A8, S100A9, I	GO:0031349~positive regulation of defense response
DDX58, FCER1A, SH2D1A, S100A8, GPR37, H	GO:0080134~regulation of response to stress*
FCER1A, S100A8, EDN1, S100A9, IL1RAP, SO	GO:0051046~regulation of secretion*
FCER1A, S100A8, CFB, S100A9, SYNCRIP, RSA	GO:0006952~defense response*
DNAJA1, SOX4, CRY1, TRIM21	GO:1903321~negative regulation of protein modifica
FCER1A, GPR183, S100A8, CFB, S100A9, EDN	GO:0048584~positive regulation of response to stimu
CDC14B, DNAJA1, SOX4, CRY1, DAXX	GO:0031396~regulation of protein ubiquitination*
FCER1A, GPR183, ACVR1B, SH2D1A, S100A8	GO:0009967~positive regulation of signal transductio
DDX58, FCER1A, GPR183, SH2D1A, CFB, LYST	GO:0002252~immune effector process*
DDX58, FCER1A, GPR183, ACVR1B, SH2D1A,	GO:0002684~positive regulation of immune system p
FCER1A, GPR183, ACVR1B, SH2D1A, S100A8	GO:0010647~positive regulation of cell communicatio
FCER1A, GPR183, ACVR1B, SH2D1A, S100A8	GO:0023056~positive regulation of signaling*
USP18, CDC14B, DTX3L, USP1, DNAJA1, SOX4	GO:0070647~protein modification by small protein co
DDX58, FCER1A, HPSE, IL1RAP, RNF128, RSA	GO:0001817~regulation of cytokine production*
DDX58, FCER1A, SH2D1A, CFB, RSAD2, TLR4,	GO:0050776~regulation of immune response*
DDX58, FCER1A, HPSE, IL1RAP, RNF128, RSA	GO:0001816~cytokine production*
FCER1A, EDN1, IL1RAP, SOX4, RSAD2, TLR4,	GO:1903530~regulation of secretion by cell*
DDX58, SH2D1A, RSAD2, TLR4	GO:0045089~positive regulation of innate immune re
DDX58, FCER1A, SH2D1A, S100A8, S100A9, I	GO:0031347~regulation of defense response*
DDX58, FCER1A, SH2D1A, CFB, RSAD2, TLR4	GO:0050778~positive regulation of immune response
FCER1A, GPR183, S100A8, S100A9, RSAD2, T	GO:0009605~response to external stimulus*
FCER1A, S100A8, EDN1, S100A9, IL1RAP, SO	GO:0046903~secretion*
FCER1A, EDN1, DNAJA1	GO:0043506~regulation of JUN kinase activity*
DDX58, DNAJA1, CRY1, DAXX	GO:0030522~intracellular receptor signaling pathway
RSAD2, TRIM21, ISG20	GO:1903901~negative regulation of viral life cycle*
ACAA2, GPR183, S100A8, IL1RN, S100A9, SY	GO:0070887~cellular response to chemical stimulus*
FCER1A, IL1RAP, RNF128	GO:0042089~cytokine biosynthetic process*
GPR183, ACVR1B, AGPAT5, BCL11B, SOX4, R	GO:0030097~hemopoiesis*
FCER1A, IL1RAP, RNF128	GO:0042107~cytokine metabolic process*
FCER1A, GPR37, USP1, EDN1, DNAJA1, CRY1	GO:0080135~regulation of cellular response to stress
DDX58, SH2D1A, RSAD2, TLR4	GO:0045088~regulation of innate immune response*
DDX58, MICALL2, IL1RAP, DNAJA1, SOX4, RS	GO:0051223~regulation of protein transport*
RSAD2, TRIM21, ISG20	GO:0048525~negative regulation of viral process*
FCER1A, ACAA2, GPR183, S100A8, S100A9, I	GO:0009966~regulation of signal transduction*
MIB1, ACVR1B, HPSE, BCL11B, ARG2, EDN1,	GO:0060429~epithelium development*
GPR183, ACVR1B, AGPAT5, BCL11B, SOX4, R	GO:0048534~hematopoietic or lymphoid organ devel
FCER1A, GPR183, S100A8, GPR37, HPSE, EDN	GO:1902533~positive regulation of intracellular signa
DNAJA1, CRY1, DAXX	GO:0030518~intracellular steroid hormone receptor
DDX58, RSAD2, TLR4	GO:0002221~pattern recognition receptor signaling p
DDX58, RSAD2, TLR4	GO:0002758~innate immune response-activating sign
DDX58, MICALL2, IL1RAP, DNAJA1, SOX4, RS	GO:0070201~regulation of establishment of protein l
RSAD2, SSB, TRIM21, ISG20	GO:0019058~viral life cycle*
GPR183, ACVR1B, AGPAT5, BCL11B, SOX4, R	GO:0002520~immune system development*

DDX58, RSAD2, TLR4	GO:0002218~activation of innate immune response*
	GO:0034097~response to cytokine
	GO:1903900~regulation of viral life cycle
	GO:0045071~negative regulation of viral genome rep
	GO:0050792~regulation of viral process
	GO:0043901~negative regulation of multi-organism p
	GO:0016032~viral process
	GO:0051607~defense response to virus
	GO:0043903~regulation of symbiosis, encompassing
	GO:0044764~multi-organism cellular process
	GO:0098542~defense response to other organism
	GO:0044419~interspecies interaction between organ
	GO:0044403~symbiosis, encompassing mutualism thr
	GO:0043900~regulation of multi-organism process
	GO:0045069~regulation of viral genome replication
	GO:0009615~response to virus
	GO:0019079~viral genome replication
	GO:0010033~response to organic substance
	GO:0002682~regulation of immune system process
	GO:0034340~response to type I interferon
	GO:0071345~cellular response to cytokine stimulus
	GO:0032439~endosome localization
	GO:0034341~response to interferon-gamma
	GO:0051707~response to other organism
	GO:0043207~response to external biotic stimulus
	GO:0009607~response to biotic stimulus
	GO:0044093~positive regulation of molecular functio
	GO:0065009~regulation of molecular function
	GO:1903320~regulation of protein modification by sn
	GO:0035455~response to interferon-alpha
	GO:0032647~regulation of interferon-alpha productio
	GO:0032607~interferon-alpha production
	GO:0007254~JNK cascade
	GO:0046328~regulation of JNK cascade
	GO:0048147~negative regulation of fibroblast prolife
	GO:0060759~regulation of response to cytokine stim
	GO:0046596~regulation of viral entry into host cell
	GO:0071357~cellular response to type I interferon
	GO:0002253~activation of immune response
	GO:0019221~cytokine-mediated signaling pathway
	GO:0008285~negative regulation of cell proliferation
	GO:0032872~regulation of stress-activated MAPK cas
	GO:0070302~regulation of stress-activated protein ki
	GO:0051403~stress-activated MAPK cascade
	GO:0045428~regulation of nitric oxide biosynthetic p
	GO:0031098~stress-activated protein kinase signaling
	GO:0071310~cellular response to organic substance
	GO:0002758~innate immune response-activating sign
	GO:0032648~regulation of interferon-beta productio
	GO:0032608~interferon-beta production

GO:0046330~positive regulation of JNK cascade
GO:1903426~regulation of reactive oxygen species bi
GO:0002757~immune response-activating signal tran
GO:0002764~immune response-regulating signaling p
GO:0051828~entry into other organism involved in sy
GO:0044409~entry into host
GO:0030260~entry into host cell
GO:0046718~viral entry into host cell
GO:0051806~entry into cell of other organism involve
GO:0032874~positive regulation of stress-activated M
GO:0070304~positive regulation of stress-activated p
GO:0032479~regulation of type I interferon productio
GO:0032606~type I interferon production
GO:0050707~regulation of cytokine secretion
GO:0071346~cellular response to interferon-gamma
GO:0044089~positive regulation of cellular compone
GO:1903409~reactive oxygen species biosynthetic pr
GO:0006915~apoptotic process
GO:0046598~positive regulation of viral entry into ho
GO:0008219~cell death
GO:0016567~protein ubiquitination
GO:0009117~nucleotide metabolic process
GO:0042976~activation of Janus kinase activity
GO:0044248~cellular catabolic process
GO:0032940~secretion by cell
GO:0006753~nucleoside phosphate metabolic proces
GO:0048145~regulation of fibroblast proliferation
GO:0001959~regulation of cytokine-mediated signali
GO:0048144~fibroblast proliferation
GO:0090501~RNA phosphodiester bond hydrolysis
GO:0035457~cellular response to interferon-alpha
GO:1903321~negative regulation of protein modifica
GO:0050790~regulation of catalytic activity
GO:0050708~regulation of protein secretion
GO:0032446~protein modification by small protein co
GO:0051701~interaction with host
GO:0008283~cell proliferation
GO:0042127~regulation of cell proliferation
GO:0012501~programmed cell death
GO:0060333~interferon-gamma-mediated signaling p
GO:0051246~regulation of protein metabolic process
GO:0002224~toll-like receptor signaling pathway
GO:0055086~nucleobase-containing small molecule m
GO:0051090~regulation of sequence-specific DNA bin
GO:0045351~type I interferon biosynthetic process
GO:0060330~regulation of response to interferon-ga
GO:0032880~regulation of protein localization
GO:0051091~positive regulation of sequence-specific
GO:0051130~positive regulation of cellular compone
GO:0032727~positive regulation of interferon-alpha p

GO:1902187~negative regulation of viral release fron
GO:0031399~regulation of protein modification proc
GO:0031647~regulation of protein stability
GO:0071396~cellular response to lipid
GO:0007166~cell surface receptor signaling pathway
GO:0006796~phosphate-containing compound meta
GO:0046777~protein autophosphorylation
GO:0001525~angiogenesis
GO:0043085~positive regulation of catalytic activity
GO:0006793~phosphorus metabolic process
GO:0051248~negative regulation of protein metaboli
GO:0009306~protein secretion
GO:0042036~negative regulation of cytokine biosyntl
GO:0060337~type I interferon signaling pathway
GO:0002313~mature B cell differentiation involved ir
GO:0006164~purine nucleotide biosynthetic process
GO:0009893~positive regulation of metabolic proces:
GO:0071229~cellular response to acid chemical
GO:0032270~positive regulation of cellular protein m
GO:0014070~response to organic cyclic compound
GO:0007249~I-kappaB kinase/NF-kappaB signaling
GO:0034655~nucleobase-containing compound catal
GO:0044265~cellular macromolecule catabolic proce
GO:0031325~positive regulation of cellular metabolic
GO:0072522~purine-containing compound biosynthe
GO:0045429~positive regulation of nitric oxide biosyr
GO:1904407~positive regulation of nitric oxide metak
GO:0002335~mature B cell differentiation
GO:0071356~cellular response to tumor necrosis fact
GO:0034654~nucleobase-containing compound biosy
GO:2000377~regulation of reactive oxygen species m

DDX58, FCER1A, MICALL2, MIB1, S100A8, EDN1, S100A9, IL1RAP, SOX4, PLTP
FCER1A, S100A8, EDN1, S100A9, IL1RAP, SOX4
FCER1A, SH2D1A, LYST, RSAD2, IL7R
MICALL2, S100A8, S100A9, SOX4, SOD2, MIB1, MAN2A1, SEMA6D, BCL11B, IL1RAP, PBX3, PLXND1
S100A8, S100A9
FCER1A, SH2D1A, RSAD2, IL7R
ACAA2, USP1, EDN1, PSIP1, DNAJA1, PPP1CC, CRY1, SOD2, TP53INP1
FCER1A, GPR183, GALNT7, S100A8, CDC14B, USP1, DTX3L, S100A9, EDN1, SOX4, DAXX, TRIM21, M
FCER1A, GPR183, GALNT7, S100A8, CDC14B, USP1, DTX3L, S100A9, EDN1, SOX4, DAXX, TRIM21, M
DDX58, GPR183, ACVR1B, S100A8, SEMA6D, GPR37, BCL11B, S100A9, EDN1, DNAJA1, TNK2, PLXN
MAN2A1, EDN1, PBX3
PLTP
FCER1A, SH2D1A, LYST, RSAD2, IL7R
FCER1A, S100A8, EDN1, S100A9, SOX4, SLC38A1, TCN1
PLTP
S100A8, S100A9, SOX4
DDX58, FCER1A, MAN2A1, ACVR1B, HPSE, EDN1, S100A9, IL1RAP, RSAD2, IL7R, PLTP
FCER1A, SH2D1A, RSAD2, IL7R
GPR37, SLC25A24, SOD2, TP53INP1

S100A8, S100A9
PSIP1, DNAJA1, TP53INP1
DNAJA1, SOX4, CRY1
FCER1A, S100A8, EDN1, S100A9, IL1RAP, SOX4, RSAD2, TLR4, CRY1
SEMA6D, EDN1, PLXND1, IL7R
GPR37, SLC25A24, PSIP1, SOD2, TP53INP1
MICALL2, MIB1, MAN2A1, SEMA6D, BCL11B, IL1RAP, SOX4, PLXND1, PBX3, ACSL6, SOD2
DDX58, GPR183, ACVR1B, SEMA6D, S100A8, EDN1, S100A9, DNAJA1, TNK2, PLXND1, TP53INP1
DDX58, GPR183, ACVR1B, SEMA6D, S100A8, EDN1, S100A9, DNAJA1, TNK2, PLXND1, TP53INP1
SH2D1A, LYST, IL7R
DDX58, FCER1A
S100A8, S100A9, SOX4
S100A8, EDN1, S100A9, SOX4
FCER1A, SH2D1A, RSAD2
ACVR1B, HPSE, BCL11B, TGM1
S100A8, BCL11B, S100A9, SOX4, RSAD2, IL7R
S100A8, S100A9, DNAJA1, SOX4, DAXX, TP53INP1
S100A8, EDN1, S100A9, SOX4
FCER1A, ACAA2, CDC14B, USP1, DTX3L, EDN1, SOX4, SOD2, GPR37, SLC25A24, DNAJA1, CRY1, TP5
S100A8, S100A9
S100A8, S100A9
DDX58, FCER1A
DDX58, FCER1A
MICALL2, S100A8, S100A9, SOX4, SOD2, MBP, MAN2A1, MIB1, SEMA6D, BCL11B, IL1RAP, PLXND1
TCIRG1, EDN1, SLC4A4
DDX58, FCER1A, HPSE, IL1RAP, RSAD2
BCL11B, SOX4, RSAD2, IL7R
CRY1, PLTP
FCER1A, SH2D1A, RSAD2
FCER1A, S100A8, S100A9
S100A8, EDN1, S100A9, SOX4
USP1, PPP1CC, CRY1, SOD2, TP53INP1
PSPC1, PPP1CC, CRY1
PLTP
SH2D1A, LYST, IL7R
S100A8, BCL11B, S100A9, SOX4, RSAD2, IL7R
ACVR1B, HPSE, BCL11B, TGM1
DDX58, FCER1A, SH2D1A, RSAD2, IL7R
MIB1, ACVR1B, HPSE, BCL11B, ARG2, EDN1, TGM1, SOX4, PLXND1
GPR183, BCL11B, SOX4, RSAD2, IL7R
PSIP1, DNAJA1, TP53INP1
PLTP
USP1, PPP1CC, CRY1, TP53INP1
DDX58, GPR183, ACVR1B, SEMA6D, S100A8, BCL11B, EDN1, S100A9, DNAJA1, TNK2, PLXND1, TP5
MICALL2, TCIRG1, PSIP1, NAP1L1, TLR4, DAXX, PLTP, TRIM21, CENPH, KCTD12
PPP1CC, CRY1
PLTP
PLTP
PPP1CC, CRY1
OSBPL8, CRY1, PLTP

GPR183, SEMA6D, S100A8, BCL11B, S100A9, PLXND1
FCER1A, GPR183, BCL11B, SOX4, RSAD2, TLR4, IL7R
GPR183, SEMA6D, S100A8, BCL11B, S100A9, PLXND1
TCIRG1, EDN1, SLC4A4
PLTP
HPSE, S100A9
HPSE, S100A9
BCL11B, TGM1
HPSE, S100A9
DDX58, FCER1A, SEMA6D, S100A8, HPSE, S100A9, PLXND1

and DR pigs. Asterisks indicate terms commonly enriched in the IR ar

	DR		
**Count_DR**	**%_DR**	**Pvalue_DR**	**Genes_DR**
22	21.35922	2.22103E-15	NMI, CFB, SOCS1, CCL8, TRIM26, RSAD2, OAS1, TLR4, STA
24	23.30097	9.22468E-11	NMI, CFB, LGALS1, SOCS1, CCL8, RSAD2, TRIM26, OAS1, T
14	13.59223	0.007946251	MICALL2, SOCS1, EDN1, RSAD2, TLR4, ANXA4, DDX58, PTK
6	5.825243	0.003358149	DDX58, PLSCR1, IRF7, CCL8, RSAD2, TLR4
17	16.50485	6.0055E-06	NMI, SOCS1, EDN1, CCL8, RSAD2, TLR4, STK3, DDX58, PLS
7	6.796117	0.033431946	EDN1, SOCS1, IL1RAP, RSAD2, SYTL2, TLR4, ANXA4
25	24.27184	1.90717E-10	NMI, CFB, SOCS1, RSAD2, TRIM26, CCL8, LGALS8, TLR4, O
3	2.912621	0.041872294	ISG15, DNAJA1, TRIM21
16	15.53398	0.003506926	CFB, LGALS1, SOCS1, EDN1, CCL8, RSAD2, TLR4, STK3, DD
5	4.854369	0.006975041	NMI, ISG15, PTK2B, DNAJA1, DAXX
11	10.67961	0.033050343	ACVR1B, TNFSF10, PTK2B, LGALS1, IRF7, EDN1, SOCS1, CC
15	14.56311	5.11276E-07	CFB, LGALS1, RSAD2, OAS1, ISG20, IFIT3, DDX58, PLSCR1,
11	10.67961	0.000601118	DDX58, PLSCR1, ACVR1B, ISG15, PTK2B, CFB, LGALS1, IRF
11	10.67961	0.049992376	ACVR1B, TNFSF10, PTK2B, LGALS1, IRF7, EDN1, SOCS1, CC
11	10.67961	0.051716033	ACVR1B, TNFSF10, PTK2B, LGALS1, IRF7, EDN1, SOCS1, CC
10	9.708738	0.004228912	USP18, NMI, ISG15, PTK2B, DTX3L, SOCS1, DNAJA1, USP2
10	9.708738	0.000198086	DDX58, NMI, ISG15, IRF7, SOCS1, IL1RAP, RNF128, RSAD2
11	10.67961	5.72783E-05	DDX58, PLSCR1, NMI, PARP9, CFB, IRF7, SOCS1, RSAD2, T
10	9.708738	0.000348481	DDX58, NMI, ISG15, IRF7, SOCS1, IL1RAP, RNF128, RSAD2
7	6.140351	0.032892685	FCER1A, EDN1, IL1RAP, SOX4, RSAD2, TLR4, CRY1
5	4.854369	0.004045983	DDX58, PLSCR1, IRF7, RSAD2, TLR4
10	9.708738	0.000364419	DDX58, PLSCR1, NMI, PARP9, IRF7, SOCS1, CCL8, RSAD2, T
7	6.796117	0.006755701	DDX58, PLSCR1, CFB, IRF7, RSAD2, TLR4, TEC
17	16.50485	0.003644783	CCL8, LGALS8, RSAD2, OAS1, TLR4, STAT1, ISG20, IFIT3, D
8	7.76699	0.066158434	EDN1, SOCS1, IL1RAP, CCL8, RSAD2, SYTL2, TLR4, ANXA4
3	2.912621	0.037228252	EDN1, DNAJA1, PAK1
4	3.883495	0.035980383	DDX58, DNAJA1, PAK1, DAXX
8	7.76699	3.38829E-08	PLSCR1, ISG15, TRIM26, RSAD2, OAS1, MX1, TRIM21, ISG
16	15.53398	0.028589243	NMI, SOCS1, IL1RN, CCL8, TLR4, UBR1, STAT1, DAXX, SOD
4	3.883495	0.005500896	NMI, IRF7, IL1RAP, RNF128
8	7.76699	0.041189856	ACVR1B, ISG15, PTK2B, LGALS1, RSAD2, ETV6, STK3, SOD
4	3.883495	0.005796414	NMI, IRF7, IL1RAP, RNF128
6	5.825243	0.045588409	PTK2B, EDN1, DNAJA1, PAK1, USP25, STK3
9	8.737864	1.58246E-06	DDX58, PLSCR1, NMI, PARP9, IRF7, SOCS1, RSAD2, TLR4, T
8	7.76699	0.015669133	DDX58, MICALL2, SOCS1, IL1RAP, DNAJA1, RSAD2, TLR4, A
8	7.76699	9.98799E-08	PLSCR1, ISG15, TRIM26, RSAD2, OAS1, MX1, TRIM21, ISG
16	15.53398	0.066522147	LGALS1, SOCS1, EDN1, IL1RN, CCL8, RSAD2, UBR1, STAT1,
9	8.737864	0.05124364	ACVR1B, TDRD7, EDN1, TGM1, PAK1, PLXND1, STAT1, AN
8	7.76699	0.05507698	ACVR1B, ISG15, PTK2B, LGALS1, RSAD2, ETV6, STK3, SOD
8	7.76699	0.056513571	TNFSF10, PTK2B, LGALS1, EDN1, SOCS1, CCL8, PAK1, STK3
3	2.912621	0.069869332	DNAJA1, PAK1, DAXX
4	3.883495	0.010092501	DDX58, IRF7, RSAD2, TLR4
4	3.883495	0.010947379	DDX58, IRF7, RSAD2, TLR4
8	7.76699	0.024493064	DDX58, MICALL2, SOCS1, IL1RAP, DNAJA1, RSAD2, TLR4, A
9	8.737864	6.08039E-06	PLSCR1, ISG15, LGALS1, TRIM26, RSAD2, OAS1, MX1, TRII
8	7.76699	0.068309908	ACVR1B, ISG15, PTK2B, LGALS1, RSAD2, ETV6, STK3, SOD

4	3.883495	0.012779698	DDX58, IRF7, RSAD2, TLR4
15	14.56311	1.11316E-07	NMI, IL1RN, SOCS1, CCL8, STAT1, TRIM21, IFIT3, IFIT2, PA
9	8.737864	1.79919E-07	PLSCR1, ISG15, LGALS1, TRIM26, RSAD2, OAS1, MX1, TRIN
6	5.825243	8.99825E-07	PLSCR1, ISG15, RSAD2, OAS1, MX1, ISG20
10	9.708738	2.47103E-06	DDX58, PLSCR1, ISG15, LGALS1, TRIM26, RSAD2, OAS1, M
8	7.76699	3.16732E-06	PLSCR1, ISG15, TRIM26, RSAD2, OAS1, MX1, TRIM21, ISG
13	12.62136	4.97901E-06	IFIT3, DDX58, IFIT2, PLSCR1, ISG15, LGALS1, RSAD2, TRIM
10	9.708738	5.09254E-06	IFIT3, DDX58, PLSCR1, IFIT2, ISG15, RSAD2, OAS1, MX1, M
10	9.708738	5.31553E-06	DDX58, PLSCR1, ISG15, LGALS1, TRIM26, RSAD2, OAS1, M
13	12.62136	5.41939E-06	IFIT3, DDX58, IFIT2, PLSCR1, ISG15, LGALS1, RSAD2, TRIM
12	11.65049	7.38519E-06	IFIT3, DDX58, PLSCR1, IFIT2, ISG15, LGALS8, RSAD2, TLR4,
13	12.62136	9.84163E-06	IFIT3, DDX58, IFIT2, PLSCR1, ISG15, LGALS1, RSAD2, TRIM
13	12.62136	9.84163E-06	IFIT3, DDX58, IFIT2, PLSCR1, ISG15, LGALS1, RSAD2, TRIM
11	10.67961	1.03519E-05	DDX58, PLSCR1, ACVR1B, ISG15, LGALS1, TRIM26, RSAD2,
6	5.825243	1.1066E-05	PLSCR1, ISG15, RSAD2, OAS1, MX1, ISG20
10	9.708738	2.28775E-05	IFIT3, DDX58, PLSCR1, IFIT2, ISG15, RSAD2, OAS1, MX1, M
6	5.825243	2.30686E-05	PLSCR1, ISG15, RSAD2, OAS1, MX1, ISG20
23	22.3301	3.78705E-05	NMI, SOCS1, IL1RN, CCL8, TLR4, UBR1, STAT1, DAXX, TRIN
15	14.56311	0.000132994	NMI, CFB, LGALS1, SOCS1, RSAD2, TLR4, DDX58, PLSCR1,
4	3.883495	0.000227095	ISG15, IRF7, STAT1, MX1
10	9.708738	0.000264502	IFIT3, IFIT2, NMI, PTK2B, IRF7, SOCS1, IL1RN, CCL8, STAT1
3	2.912621	0.000269814	MX1, ANKFY1, MYO5B
5	4.854369	0.000327911	NMI, SOCS1, CCL8, STAT1, TRIM21
12	11.65049	0.000349881	IFIT3, DDX58, PLSCR1, IFIT2, ISG15, LGALS8, RSAD2, TLR4,
12	11.65049	0.00035617	IFIT3, DDX58, PLSCR1, IFIT2, ISG15, LGALS8, RSAD2, TLR4,
12	11.65049	0.000634745	IFIT3, DDX58, PLSCR1, IFIT2, ISG15, LGALS8, RSAD2, TLR4,
14	13.59223	0.000739933	EDN1, SOCS1, MED25, CCL8, TRIM26, STAT1, TRIM21, STK
19	18.4466	0.001078447	HSP90AA1, SOCS1, EDN1, MED25, CCL8, TRIM26, STAT1,
6	5.825243	0.001617345	NMI, ISG15, PTK2B, DNAJA1, DAXX, TRIM21
3	2.912621	0.001970875	IFIT3, IFIT2, MX2
3	2.912621	0.002398288	DDX58, NMI, IRF7
3	2.912621	0.002865336	DDX58, NMI, IRF7
5	4.854369	0.003338912	PTK2B, EDN1, DNAJA1, PAK1, STK3
5	4.854369	0.003752056	PTK2B, EDN1, DNAJA1, PAK1, STK3
3	2.912621	0.003916173	MORC3, MED25, SOD2
4	3.883495	0.003919678	PARP9, IRF7, SOCS1, IL1RN
3	2.912621	0.004498893	LGALS1, TRIM26, TRIM21
3	2.912621	0.005119109	IRF7, STAT1, MX1
6	5.825243	0.005178673	DDX58, CFB, IRF7, RSAD2, TLR4, TEC
7	6.796117	0.006015309	NMI, PTK2B, IRF7, SOCS1, IL1RN, CCL8, STAT1
8	7.76699	0.006362846	IFIT3, PTK2B, MORC3, MED25, PAK1, STAT1, STK3, SOD2
5	4.854369	0.00719449	PTK2B, EDN1, DNAJA1, PAK1, STK3
5	4.854369	0.007418462	PTK2B, EDN1, DNAJA1, PAK1, STK3
5	4.854369	0.008360342	PTK2B, EDN1, DNAJA1, PAK1, STK3
3	2.912621	0.008764572	HSP90AA1, PTK2B, SOD2
5	4.854369	0.009915176	PTK2B, EDN1, DNAJA1, PAK1, STK3
15	14.56311	0.010554133	NMI, SOCS1, IL1RN, CCL8, TLR4, UBR1, STAT1, DAXX, IFIT3
4	3.883495	0.010947379	DDX58, IRF7, RSAD2, TLR4
3	2.912621	0.012304059	DDX58, NMI, IRF7
3	2.912621	0.012304059	DDX58, NMI, IRF7

4	3.883495	0.012779698	PTK2B, EDN1, PAK1, STK3
3	2.912621	0.013271403	HSP90AA1, PTK2B, SOD2
5	4.854369	0.013563045	DDX58, IRF7, RSAD2, TLR4, TEC
5	4.854369	0.015304813	DDX58, IRF7, RSAD2, TLR4, TEC
3	2.912621	0.01745652	LGALS1, TRIM26, TRIM21
3	2.912621	0.01745652	LGALS1, TRIM26, TRIM21
3	2.912621	0.01745652	LGALS1, TRIM26, TRIM21
3	2.912621	0.01745652	LGALS1, TRIM26, TRIM21
3	2.912621	0.01745652	LGALS1, TRIM26, TRIM21
4	3.883495	0.017509768	PTK2B, EDN1, PAK1, STK3
4	3.883495	0.018087832	PTK2B, EDN1, PAK1, STK3
3	2.912621	0.019732102	DDX58, NMI, IRF7
3	2.912621	0.022124917	DDX58, NMI, IRF7
4	3.883495	0.022430694	SOCS1, IL1RAP, TLR4, ANXA4
3	2.912621	0.023364192	NMI, CCL8, STAT1
6	5.825243	0.025184348	PTK2B, EDN1, IL1RAP, MED25, TLR4, PAK1
3	2.912621	0.025926324	HSP90AA1, PTK2B, SOD2
12	11.65049	0.026199418	IFIT3, PLSCR1, ACVR1B, TNFSF10, PTK2B, LGALS1, DNAJA1
2	1.941748	0.026851313	LGALS1, TRIM21
13	12.62136	0.030688774	IFIT3, ACVR1B, PLSCR1, TNFSF10, PTK2B, LGALS1, DNAJA1
7	6.796117	0.031509034	NMI, ISG15, PTK2B, DTX3L, SOCS1, DNAJA1, TRIM21
7	6.796117	0.033431946	TCIRG1, PTK2B, HK3, EDN1, UPP1, CNP, OAS1
2	1.941748	0.033452213	PTK2B, SOCS1
12	11.65049	0.034193677	TCIRG1, USP18, ISG15, PTK2B, CNDP2, RNASET2, UPP1, A[illegible]
8	7.76699	0.034852493	EDN1, SOCS1, IL1RAP, CCL8, RSAD2, SYTL2, TLR4, ANXA4
7	6.796117	0.035430112	TCIRG1, PTK2B, HK3, EDN1, UPP1, CNP, OAS1
3	2.912621	0.035727598	MORC3, MED25, SOD2
3	2.912621	0.037228252	IRF7, SOCS1, IL1RN
3	2.912621	0.037228252	MORC3, MED25, SOD2
3	2.912621	0.038752842	RNASE4, RNASET2, ISG20
2	1.941748	0.040008932	IFIT3, IFIT2
3	2.912621	0.041872294	ISG15, DNAJA1, TRIM21
12	11.65049	0.042085233	PLSCR1, TNFSF10, HSP90AA1, PTK2B, EDN1, SOCS1, DNAJ[illegible]
5	4.854369	0.042556145	SOCS1, IL1RAP, RSAD2, TLR4, ANXA4
7	6.796117	0.043723956	NMI, ISG15, PTK2B, DTX3L, SOCS1, DNAJA1, TRIM21
3	2.912621	0.045082931	LGALS1, TRIM26, TRIM21
12	11.65049	0.045275982	IFIT3, PTK2B, MORC3, EDN1, TGM1, TGFBI, MED25, PAK1,
11	10.67961	0.046339035	IFIT3, PTK2B, MORC3, EDN1, TGM1, MED25, PAK1, STAT1,
12	11.65049	0.046477003	IFIT3, PLSCR1, ACVR1B, TNFSF10, PTK2B, LGALS1, DNAJA1
2	1.941748	0.046521762	NMI, STAT1
16	15.53398	0.049290253	NMI, SOCS1, EDN1, CCL8, STAT1, DAXX, TRIM21, STK3, TN[illegible]
3	2.912621	0.050063393	IRF7, RSAD2, TLR4
7	6.796117	0.051073521	TCIRG1, PTK2B, HK3, EDN1, UPP1, CNP, OAS1
5	4.854369	0.05180793	IL1RAP, TRIM26, ANXA4, STK3, TRIM21
2	1.941748	0.052990992	NMI, IRF7
2	1.941748	0.052990992	PARP9, SOCS1
8	7.76699	0.05507698	DDX58, MICALL2, SOCS1, IL1RAP, DNAJA1, RSAD2, TLR4, [illegible]
4	3.883495	0.058159533	IL1RAP, TRIM26, STK3, TRIM21
9	8.737864	0.059395694	MICALL2, TNFSF10, PTK2B, EDN1, IL1RAP, MED25, TLR4, [illegible]
2	1.941748	0.05941691	DDX58, IRF7

2	1.941748	0.05941691	TRIM26, TRIM21
12	11.65049	0.060806737	NMI, ISG15, PTK2B, EDN1, SOCS1, DNAJA1, CCL8, PAK1, D
4	3.883495	0.0636693	MORC3, RNF128, STK3, TRIM21
5	4.854369	0.065441638	PTK2B, DNAJA1, TLR4, PAK1, DAXX
15	14.56311	0.065861906	NMI, SOCS1, EDN1, IL1RN, CCL8, TLR4, STAT1, ANXA4, STK
17	16.50485	0.067033674	TCIRG1, SOCS1, EDN1, UPP1, CCL8, OAS1, CNP, STK3, PPA
4	3.883495	0.067087827	ACVR1B, PTK2B, PAK1, TEC
5	4.854369	0.067158483	PTK2B, EDN1, TGFBI, PLXND1, STAT1
8	7.76699	0.06776808	PLSCR1, TNFSF10, PTK2B, EDN1, SOCS1, CCL8, PAK1, STAT
17	16.50485	0.068271866	TCIRG1, SOCS1, EDN1, UPP1, CCL8, OAS1, CNP, STK3, PPA
8	7.76699	0.072739681	NMI, ISG15, EDN1, SOCS1, RNF128, DNAJA1, USP25, TRIM
5	4.854369	0.073351602	SOCS1, IL1RAP, RSAD2, TLR4, ANXA4
2	1.941748	0.078437647	NMI, RNF128
2	1.941748	0.078437647	IRF7, STAT1
2	1.941748	0.078437647	PTK2B, LGALS1
4	3.883495	0.080320996	TCIRG1, PTK2B, EDN1, OAS1
17	16.50485	0.081178471	HSP90AA1, NMI, SOCS1, EDN1, MED25, CCL8, STAT1, STK
3	2.912621	0.083590347	PTK2B, SOCS1, UBR1
10	9.708738	0.085894113	TNFSF10, NMI, PTK2B, EDN1, SOCS1, CCL8, PAK1, STAT1,
6	5.825243	0.086159448	DDX58, USP18, DNAJA1, PAK1, STAT1, DAXX
4	3.883495	0.087983652	TNFSF10, LGALS1, TIFA, STAT1
4	3.883495	0.087983652	RNASET2, UPP1, CNP, ISG20
8	7.76699	0.088305502	TCIRG1, USP18, ISG15, PTK2B, RNASET2, UBR1, USP25, IS
16	15.53398	0.089052405	HSP90AA1, NMI, SOCS1, EDN1, MED25, CCL8, STAT1, STK
4	3.883495	0.090604779	TCIRG1, PTK2B, EDN1, OAS1
2	1.941748	0.090906776	HSP90AA1, SOD2
2	1.941748	0.090906776	HSP90AA1, SOD2
2	1.941748	0.090906776	PTK2B, LGALS1
3	2.912621	0.091771039	PTK2B, CCL8, STAT1
20	19.41748	0.096215537	TCIRG1, ETV7, NMI, EDN1, MED25, TRIM26, OAS1, STAT1,
3	2.912621	0.098054691	HSP90AA1, PTK2B, SOD2

., ACSL6

MAN2A1, ACVR1B, VRK1, USP18, GPR37, B3GALNT1, TGM1, DNAJA1, PRKACB, TNK2, CRY1
MAN2A1, ACVR1B, VRK1, USP18, GPR37, B3GALNT1, TGM1, DNAJA1, PRKACB, TNK2, CRY1
D1, TP53INP1

3INP1

, PBX3, ACSL6

3INP1

ıd the DR pigs.

T1, TRIM21, ISG20, DDX58, PLSCR1, PARP9, ISG15, PTK2B, IRF7, LYST, TRAFD1, MX1, MX2, TEC
LR4, STAT1, TRIM21, ISG20, DDX58, PLSCR1, TNFSF10, PARP9, ISG15, PTK2B, IRF7, LYST, TRAFD1, MX1, M
(2B, IL1RAP, DNAJA1, SYTL2, MX1, MX2, ANKFY1

CR1, PARP9, PTK2B, IRF7, DNAJA1, PAK1, TRAFD1, USP25, TEC

AS1, STAT1, TRIM21, ISG20, IFIT3, DDX58, PLSCR1, IFIT2, ISG15, PARP9, PTK2B, LYST, IRF7, TRAFD1, MX1,

(58, PLSCR1, ACVR1B, TNFSF10, PTK2B, IRF7, PAK1, TEC

L8, RSAD2, PAK1, STK3
IFIT2, ISG15, PTK2B, LYST, IRF7, MX1, MX2
7, RSAD2, TLR4, TEC
L8, RSAD2, PAK1, STK3
L8, RSAD2, PAK1, STK3
5, DAXX, TRIM21
, TLR4, ANXA4
R4, TRAFD1, TEC
, TLR4, ANXA4

LR4, TRAFD1

DX58, PLSCR1, IFIT2, ISG15, PTK2B, PLXND1, MX1, MX2, TEC

20
2, IFIT3, IFIT2, PTK2B, IRF7, DNAJA1, PAK1, MX1

2

RAFD1
NXA4
20
STK3, PLSCR1, ACVR1B, TNFSF10, PTK2B, IRF7, DNAJA1, PAK1
XA4, STK3
2

NXA4
M21, ISG20
2

RP9, ISG15, PTK2B, IRF7, XAF1, MX1, MX2
/I21, ISG20

X1, TRIM21, ISG20
20
26, OAS1, MX1, MX2, TRIM21, ISG20
IX2, ISG20
X1, TRIM21, ISG20
26, OAS1, MX1, MX2, TRIM21, ISG20
OAS1, MX1, MX2, ISG20
26, OAS1, MX1, MX2, TRIM21, ISG20
26, OAS1, MX1, MX2, TRIM21, ISG20
OAS1, MX1, TRIM21, ISG20

IX2, ISG20

/I21, IFIT3, DDX58, IFIT2, USP18, PARP9, ISG15, PTK2B, IRF7, DNAJA1, XAF1, PAK1, USP25, MX1, MX2
ACVR1B, PARP9, ISG15, PTK2B, IRF7, TRAFD1, TEC

, MX1

OAS1, MX1, MX2, ISG20
OAS1, MX1, MX2, ISG20
OAS1, MX1, MX2, ISG20
K3, PLSCR1, TNFSF10, PTK2B, IL1RAP, PAK1, PLXND1
ANXA4, TRIM21, STK3, SOD2, PLSCR1, TNFSF10, PTK2B, IL1RAP, DNAJA1, SYTL2, PAK1, PLXND1

, IFIT2, PTK2B, IRF7, DNAJA1, PAK1, MX1

, XAF1, STAT1, ETV6, STK3, SOD2

, XAF1, STAT1, ETV6, DAXX, STK3, SOD2

SRGL1, CNP, UBR1, USP25, ISG20

A1, CCL8, SYTL2, PAK1, STAT1, SOD2

STAT1, ETV6, STK3, SOD2
, STK3, SOD2, TEC
, XAF1, STAT1, ETV6, STK3, SOD2

FSF10, ISG15, PTK2B, RNF128, DNAJA1, PAK1, USP25, TEC

ANXA4

PAK1, ANKFY1

AXX, STK3, TRIM21, TEC

K3, ACVR1B, TNFSF10, PTK2B, IRF7, PLXND1, TEC
1, ACVR1B, MORC3, PTK2B, HK3, DNAJA1, SYTL2, PAK1, TEC

T1
1, ACVR1B, MORC3, PTK2B, HK3, DNAJA1, SYTL2, PAK1, TEC
M21

3, SOD2, DDX58, PLSCR1, ACVR1B, TNFSF10, PTK2B, IRF7, PAK1, TEC

STK3, TEC

G20
3, SOD2, DDX58, PLSCR1, TNFSF10, PTK2B, IRF7, PAK1, TEC

, DAXX, ANXA4, TRIM21, STK3, SOD2, DDX58, PLSCR1, ACVR1B, PTK2B, IRF7, IL1RAP, ETV6

MX2, TEC

MX2, TEC

Comparative GO distribution of DEGs involved in the molecular function (M

IR

Term_IR	Count_DR	%_DR	Pvalue_DR	Genes_DR
GO:0046872~metal ion binding*	25	21.92982	0.061649169	MICALL2, S100A8, SPOCK2, ZNI
GO:0043167~ion binding*	26	22.80702	0.061751911	BCAT1, MICALL2, S100A8, SPO(
GO:0043169~cation binding*	25	21.92982	0.06819427	MICALL2, S100A8, SPOCK2, ZNI
GO:0008270~zinc ion binding*	11	9.649123	0.083715872	DDX58, MICALL2, MIB1, MAN2
GO:0050543~icosatetraenoic acid bin	2	1.754386	0.030522386	S100A8, S100A9
GO:0050544~arachidonic acid binding	2	1.754386	0.030522386	S100A8, S100A9
GO:0050542~icosanoid binding	2	1.754386	0.030522386	S100A8, S100A9
GO:0008525~phosphatidylcholine tra	2	1.754386	0.038008012	PLTP
GO:0036041~long-chain fatty acid bir	2	1.754386	0.038008012	S100A8, S100A9
GO:0005504~fatty acid binding	2	1.754386	0.095872611	S100A8, S100A9

MF) of IR and DR pigs. Asterisks indicate terms commonly enriched in the IF

DR

Term_DR	Count_DR	%_DR	Pvalue_DR	Genes_DR
GO:0046872~metal ion binding*	26	25.24272	0.014807747	RNASEL, CNDP2, ZNFX1
GO:0043167~ion binding*	26	25.24272	0.026724658	RNASEL, CNDP2, ZNFX1
GO:0043169~cation binding*	26	25.24272	0.016808291	RNASEL, CNDP2, ZNFX1
GO:0008270~zinc ion binding*	14	13.59223	0.003917731	MICALL2, ZNFX1, CNDF
GO:0004540~ribonuclease activity	4	3.883495	0.008633639	RNASEL, RNASE4, RNAS
GO:0046914~transition metal ion bindin	15	14.56311	0.009255065	MICALL2, ZNFX1, CNDF
GO:0097367~carbohydrate derivative bi	19	18.4466	0.015481558	RNASEL, HSP90AA1, CC
GO:0008242~omega peptidase activity	2	1.941748	0.028365696	GGH, ASRGL1
GO:0005536~glucose binding	2	1.941748	0.042249186	SLC2A3, HK3
GO:0030246~carbohydrate binding	4	3.883495	0.047322123	SLC2A3, LGALS1, HK3,
GO:0005524~ATP binding	13	12.62136	0.049072178	DDX58, TOR3A, ACVR1
GO:0032559~adenyl ribonucleotide binc	13	12.62136	0.054599424	DDX58, TOR3A, ACVR1
GO:0030554~adenyl nucleotide binding	13	12.62136	0.056303505	DDX58, TOR3A, ACVR1
GO:0035639~purine ribonucleoside tripl	15	14.56311	0.061417412	RNASEL, HSP90AA1, O/
GO:0001883~purine nucleoside binding	15	14.56311	0.064106289	RNASEL, HSP90AA1, O/
GO:0032550~purine ribonucleoside binc	15	14.56311	0.064106289	RNASEL, HSP90AA1, O/
GO:0032549~ribonucleoside binding	15	14.56311	0.064790939	RNASEL, HSP90AA1, O/
GO:0001882~nucleoside binding	15	14.56311	0.066175239	RNASEL, HSP90AA1, O/
GO:0032555~purine ribonucleotide bind	15	14.56311	0.070448974	RNASEL, HSP90AA1, O/
GO:0017076~purine nucleotide binding	15	14.56311	0.07302638	RNASEL, HSP90AA1, O/
GO:0004518~nuclease activity	4	3.883495	0.073885549	RNASEL, RNASE4, RNAS
GO:0032553~ribonucleotide binding	15	14.56311	0.075666505	RNASEL, HSP90AA1, O/
GO:0005518~collagen binding	2	1.941748	0.076106933	TGFBI, PAK1
GO:0008238~exopeptidase activity	3	2.912621	0.076909987	CNDP2, GGH, ASRGL1
GO:0004702~receptor signaling protein	3	2.912621	0.087505491	ACVR1B, PAK1, STK3
GO:0016462~pyrophosphatase activity	8	7.76699	0.090862025	DDX58, TCIRG1, TOR3/
GO:0016773~phosphotransferase activit	8	7.76699	0.090862025	ACVR1B, RNASEL, PTK2
GO:0016818~hydrolase activity, acting c	8	7.76699	0.091543534	DDX58, TCIRG1, TOR3/
GO:0016817~hydrolase activity, acting c	8	7.76699	0.096395913	DDX58, TCIRG1, TOR3/

R and the DR pigs.

1, RSAD2, OAS1, ISG20, MORC3, DMD, TGM1, RNF128, XAF1, MICALL2, DTX3L, TRIM26, PHF11, UBR1, Al
1, RSAD2, OAS1, ISG20, MORC3, DMD, TGM1, RNF128, XAF1, MICALL2, DTX3L, TRIM26, PHF11, UBR1, Al
1, RSAD2, OAS1, ISG20, MORC3, DMD, TGM1, RNF128, XAF1, MICALL2, DTX3L, TRIM26, PHF11, UBR1, Al
P2, DTX3L, TRIM26, PHF11, UBR1, TRIM21, DDX58, MORC3, DMD, RNF128, WHSC1L1, XAF1
SET2, ISG20
P2, DTX3L, TRIM26, PHF11, UBR1, TRIM21, SOD2, DDX58, MORC3, DMD, WHSC1L1, RNF128, XAF1
CL8, OAS1, TLR4, STK3, DDX58, TOR3A, TNFAIP6, ACVR1B, PTK2B, HK3, DMD, DNAJA1, PAK1, MX1, MX2,

LGALS8
B, RNASEL, HSP90AA1, PTK2B, HK3, DNAJA1, OAS1, PAK1, STK3, MYO5B, TEC
B, RNASEL, HSP90AA1, PTK2B, HK3, DNAJA1, OAS1, PAK1, STK3, MYO5B, TEC
B, RNASEL, HSP90AA1, PTK2B, HK3, DNAJA1, OAS1, PAK1, STK3, MYO5B, TEC
AS1, STK3, DDX58, TOR3A, ACVR1B, PTK2B, HK3, DNAJA1, PAK1, MX1, MX2, MYO5B, TEC
AS1, STK3, DDX58, TOR3A, ACVR1B, PTK2B, HK3, DNAJA1, PAK1, MX1, MX2, MYO5B, TEC
AS1, STK3, DDX58, TOR3A, ACVR1B, PTK2B, HK3, DNAJA1, PAK1, MX1, MX2, MYO5B, TEC
AS1, STK3, DDX58, TOR3A, ACVR1B, PTK2B, HK3, DNAJA1, PAK1, MX1, MX2, MYO5B, TEC
AS1, STK3, DDX58, TOR3A, ACVR1B, PTK2B, HK3, DNAJA1, PAK1, MX1, MX2, MYO5B, TEC
AS1, STK3, DDX58, TOR3A, ACVR1B, PTK2B, HK3, DNAJA1, PAK1, MX1, MX2, MYO5B, TEC
AS1, STK3, DDX58, TOR3A, ACVR1B, PTK2B, HK3, DNAJA1, PAK1, MX1, MX2, MYO5B, TEC
SET2, ISG20
AS1, STK3, DDX58, TOR3A, ACVR1B, PTK2B, HK3, DNAJA1, PAK1, MX1, MX2, MYO5B, TEC

A, HSP90AA1, MX1, MX2, MYO5B, PPA1
2B, HK3, CCL8, PAK1, STK3, TEC
A, HSP90AA1, MX1, MX2, MYO5B, PPA1
A, HSP90AA1, MX1, MX2, MYO5B, PPA1

NXA4, TRIM21, STK3, SOD2, PPA1, DDX58, PLSCR1, WHSC1L1, SYTL2, ANKFY1
NXA4, TRIM21, STK3, SOD2, PPA1, DDX58, PLSCR1, WHSC1L1, SYTL2, ANKFY1
NXA4, TRIM21, STK3, SOD2, PPA1, DDX58, PLSCR1, WHSC1L1, SYTL2, ANKFY1

MYO5B, TEC

GO distribution of DEGs involved in the cellular component (CC) of

Category	Term	Count	%	PValue
GOTERM_CC_FAT	GO:0016605~PML body	3	2.912621	0.037943
GOTERM_CC_FAT	GO:0048770~pigment granule	3	2.912621	0.03965
GOTERM_CC_FAT	GO:0042470~melanosome	3	2.912621	0.03965
GOTERM_CC_FAT	GO:0070062~extracellular exosome	18	17.47573	0.077903
GOTERM_CC_FAT	GO:1903561~extracellular vesicle	18	17.47573	0.0828
GOTERM_CC_FAT	GO:0043230~extracellular organelle	18	17.47573	0.083185
GOTERM_CC_FAT	GO:0005576~extracellular region	26	25.24272	0.08476

DR pigs

Genes	List Total	Pop Hits	Pop Total
MORC3, DAXX, ISG20	68	41	8897
HSP90AA1, SYTL2, CNP	68	42	8897
HSP90AA1, SYTL2, CNP	68	42	8897
SLCO4C1, RNASE4, LGALS1, GGH, LGALS8, CNP, ANXA4, PPA1, TOR3A, ACVI	68	1590	8897
SLCO4C1, RNASE4, LGALS1, GGH, LGALS8, CNP, ANXA4, PPA1, TOR3A, ACVI	68	1603	8897
SLCO4C1, RNASE4, LGALS1, GGH, LGALS8, CNP, ANXA4, PPA1, TOR3A, ACVI	68	1604	8897
EDN1, CCL8, CNP, OAS1, ACVR1B, TOR3A, NANS, ISG15, RNASET2, TGFBI, T	68	2572	8897

Fold Enrich	Bonferroni	Benjamini	FDR
9.573529	0.996473	0.996473	36.85624
9.345588	0.997279	0.947837	38.17523
9.345588	0.997279	0.947837	38.17523
1.481188	0.999993	0.980689	61.86345
1.469175	0.999997	0.957349	64.20243
1.468259	0.999997	0.920819	64.3807
1.322626	0.999998	0.884118	65.10108

Pathway in the DR pigs

Category	Term	Count	%	PValue
KEGG_PATHWAY	ssc05164:Influenza A	9	8.737864	6.12E-06
KEGG_PATHWAY	ssc05162:Measles	7	6.796117	1.35E-04
KEGG_PATHWAY	ssc05168:Herpes simplex infection	6	5.825243	0.004774
KEGG_PATHWAY	ssc04623:Cytosolic DNA-sensing pathway	4	3.883495	0.005995
KEGG_PATHWAY	ssc05160:Hepatitis C	5	4.854369	0.007275
KEGG_PATHWAY	ssc05161:Hepatitis B	5	4.854369	0.013181
KEGG_PATHWAY	ssc04062:Chemokine signaling pathway	5	4.854369	0.020727
KEGG_PATHWAY	ssc04060:Cytokine-cytokine receptor interaction	5	4.854369	0.045098
KEGG_PATHWAY	ssc04622:RIG-I-like receptor signaling pathway	3	2.912621	0.064589

Genes	List To	Pop Hits	Pop Total	Fold Enrich	Bonferroni
DDX58, RNASEL, TNFSF10, IRF7, RSAD2, TLR4, OAS1, STAT	44	167	7030	8.610506	7.10E-04
DDX58, TNFSF10, IRF7, TLR4, OAS1, STAT1, MX1	44	132	7030	8.472796	0.015514
DDX58, RNASEL, IRF7, OAS1, STAT1, DAXX	44	182	7030	5.267233	0.425962
DDX58, IRF7, TREX1, ZBP1	44	61	7030	10.4769	0.502177
DDX58, RNASEL, IRF7, OAS1, STAT1	44	127	7030	6.290265	0.571271
DDX58, PTK2B, IRF7, TLR4, STAT1	44	151	7030	5.290488	0.785451
PTK2B, CCL8, GNB4, PAK1, STAT1	44	173	7030	4.617709	0.911932
ACVR1B, TNFSF10, IL1RAP, CCL8, IL13RA1	44	221	7030	3.614768	0.995266
DDX58, ISG15, IRF7	44	68	7030	7.048797	0.999567

Benjamini	FDR
7.10E-04	0.006985
0.007788	0.153666
0.168912	5.312784
0.16002	6.630135
0.155817	7.992231
0.226273	14.04834
0.29326	21.25538
0.487845	40.93197
0.577087	53.31581
